# Projecting an ultra-strongly-coupled system in a non-energy-eigenbasis with a driven nonlinear resonator

**DOI:** 10.1038/s41598-019-56866-1

**Published:** 2020-02-04

**Authors:** Suguru Endo, Yuichiro Matsuzaki, Kosuke Kakuyanagi, Shiro Saito, Neill Lambert, Franco Nori

**Affiliations:** 1 Theoretical Quantum Physics Laboratory, Cluster for Pioneering Research, RIKEN, 351-0198 Wako-shi, Japan; 2 National Institute of Advanced Industrial Science and Technology AIST, Tsukuba Central 2, Umezono 1-1-1, Tsukuba, Ibaraki 305-8568 Japan; 3 NTT Basic Research Laboratories, NTT Corporation, 3-1 Morinosato-Wakamiya, Atsugi, Kanagawa 243-0198 Japan; 4 Department of Physics, The University of Michigan, Ann Arbor, MI 48109-1040 USA

**Keywords:** Physics, Quantum physics, Quantum mechanics

## Abstract

We explore the problem of projecting the ground-state of an ultra-strong-coupled circuit-QED system into a non-energy-eigenstate. As a measurement apparatus we consider a nonlinear driven resonator. We find that the post-measurement state of the nonlinear resonator exhibits a large correlation with the post-measurement state of the ultra-strongly coupled system even when the coupling between measurement device and system is much smaller than the energy scales of the system itself. While the projection is imperfect, we argue that because of the strong nonlinear response of the resonator it works in a practical regime where a linear measurement apparatus would fail.

## Introduction

A quantum measurement typically projects the quantum state of a system into an eigenstate of a measured observable $\hat{A}$. In quantum measurement theory, the measurement apparatus interacts with the target system due to an interaction Hamiltonian ${\hat{H}}_{I}=J\hat{A}\otimes \hat{B}$, where $\hat{B}$ denotes an operator of the apparatus and $J$ denotes a coupling strength^1,2^. After allowing the system and apparatus to interact for some time, they become strongly correlated. A subsequent measurement on the apparatus itself implements the desired projection of the target system, and the readout of the apparatus is associated with the eigenvalues of the system observable $\hat{A}$. The relationship between the operator $\hat{A}$ and the natural Hamiltonian describing the system is typically important. For example, to realize a quantum non-demolition (QND) measurement, a minimal condition is that the observable $\hat{A}$ should commute with the target system Hamiltonian^2–6^.

Alternatively, as a means of control, in addition to measurement, it is interesting to consider strongly non-QND measurements^7–9^. Surprisingly, such non-energy-eigenbasis projective measurements are sometimes not straightforward. Ideally, if the system observable $\hat{A}$ to be measured does not commute with the system Hamiltonian, $\hat{A}$ must have matrix elements to induce transitions between the energy eigenstates. Importantly, however, if the coupling strength $J$ is much smaller than the energy-level separation of the system, such transition matrix elements disappear under a rotating-wave approximation^10^ (see Appendix A for details), and we cannot project the system into the eigenbasis of $\hat{A}$; the system remains only minimally perturbed by the measurement apparatus, and as such stays in its energy eigenbasis.

On the other hand, in the ideal case, if the coupling between the system and apparatus is much larger than the system energy, one can perform a projective measurement much faster than the typical time scale of the system, hence realizing the desired non-energy eigenbasis measurements^11–13^. However, if these energy scales are comparable, the dynamics, and the subsequent quantum measurement process, become much more complicated than the cases described above. Understanding the interaction between the apparatus and system, and their dynamics, is important not only for explaining the mechanism of quantum projective measurements away from ideal parameter regimes, but also to achieve a higher level of control over quantum systems.

The ultra-strong coupling (USC) regime between atoms and light^14,15^ is especially attractive and practical areas in which to explore the possibility of non-energy eigenbasis measurements. This is because the ground state of this system exhibits non-trivial entanglement between the atom and photons, and virtual excitations, which are difficult to probe with energy eigenbasis measurements alone. If one cannot dynamically control the coupling strength between light and matter due to experimental limitations, being able to measure and control the non-eigenstate structure of such systems would allow one to more carefully validate the properties of the system, choose between competing models of the same physical phenomena^16^, and manipulate them for quantum information purposes.

In addition, when the coupling strength between light and matter becomes extremely strong (also known as ultra-strong coupling (USC) limit), so that it is comparable with the cavity resonance frequency, it is predicted that a new ground state will emerge^17–35^. Such a regime was recently experimentally demonstrated^36–38^, and has the potential to be realized in a large variety of devices^14,15^, including hybrid systems with artificial enhancement of coupling and collective effects^39–47^. Interestingly, non-eigenbasis measurements on a ground state of a system in the USC regime could potentially be used to induce an optical cat state, which is itself a resource for quantum information processing^22,23^.

Given such potential benefits, and open problems to be solved, this ultra-strongly-coupled system is attractive as an example with which to investigate the problem of non-energy eigenbasis measurements. Although there are several previous works studying how to detect virtual-excitations in the ground state in an ultra-strongly-coupled system^17,19–21,25,48,49^, here we focus only on how to perform non-energy eigenbasis measurements on the ground state of such a system. This would allow us, for example, to gain some information of the weights of the different components in the ground-state, as well as projecting the system into superspositions of eigenstates.

In this paper, we specifically analyze the full dynamics of an ultra-strongly coupled system interacting with a nonlinear measurement apparatus, in the above described situation (where the measured system observable does not commute with the system Hamiltonian). We evaluate the dynamics of the measurement apparatus during the interaction period, the back-action of the measurements on the system, and the correlations that build up between the system and the apparatus.

Such properties are typically studied when one tries to examine in detail a quantum measurement process^50–53^. Although there exist theoretical proposals to use a detector that continuously monitors the system^12^, here we consider a binary-outcome measurement performed on the measurement apparatus *after* the measurement apparatus and system have been allowed to interact. Such a binary-outcome measurement is understood to induce a strong correlation with the system^54,55^.

While linear resonators are used as a standard method for quantum measurement in cavity quantum electrodynamics and circuit quantum electrodynamics, in some cases a nonlinearity has been employed to improve qubit readout^4–6,54,56,57^. Due to the bifurcation effect, the state of the nonlinear resonator becomes highly sensitive to the state of the system, which enables one to implement a high-visibility readout.

Here, full numerical modelling and a low-energy approximation allow us to understand how a driven nonlinear resonator interacting with the ultra-strongly-coupled system operates, albeit with limited fidelity, in a regime where one might expect it not to work at all. This is surprising because, although the coupling between the nonlinear measurement device and the ultra-strongly-coupled system is weak compared to system energy scales, we find that, on reasonable time-scales, a strong correlation between them builds up, leading to a partial non-energy-eigenbasis projection of the USC system. We find that such a non-trivial strong correlation comes from the AC Stark effect induced by the driven nonlinear resonator.

To understand better this build up of correlations, we evaluate how quantum correlations, such as entanglement and quantum discord, are generated between system and measurement device during their interaction period. These additional results reveal the conditions neccessary for a non-energy eigenbasis measurement to be realized with this system.

The remainder of this paper is organized as follows. First, we introduce the USC system and its ground state. Second, we discuss the nature of interaction between the nonlinear resonator and the USC system, and we introduce a coarse-graining measurement of the nonlinear resonator itself. Third, we present numerical results to show how a strong correlation arises, even in a parameter regime where the coupling strength may be incorrectly considered to be negligible. A two-level approximation of the USC system allows us to understand how to gain information on the ground-state structure, what it means for the USC system to project onto superpositions of eigenstates, and the relationship between the measurement process and the AC-stark effect.

## Ultra-strong Coupling Between Light and Matter

The Hamiltonian of light in a single-mode cavity ultra-strongly coupled to matter (where the matter is well described by a two-level system) is, in its simplest form, given by the Rabi model^58^

$${\hat{H}}_{{\rm{Rabi}}}=\frac{{\omega }_{{\rm{q}}}}{2}{\hat{\sigma }}_{x}+g(\hat{a}+{\hat{a}}^{\dagger }){\hat{\sigma }}_{z}+{\omega }_{{\rm{r}}}{\hat{a}}^{\dagger }\hat{a},$$

where $\hat{a}$ (${\hat{a}}^{\dagger }$) is an annihilation (creation) operator for the single-mode cavity/resonator, ${\omega }_{{\rm{q}}}$ (${\omega }_{{\rm{r}}}$) denotes the qubit (resonator) frequency, and $g$ is the coupling strength between resonator (light) and qubit (matter).

Recall that, when the matter is in the form of a superconducting flux qubit, as in the recent ultra-strong coupling experiments in^36–38^, ${\hat{\sigma }}_{z}=|L\rangle \langle L|-|R\rangle \langle R|$ is diagonal in the persistent-current basis of $L$ and $R$ of the superconducting flux qubit. Here, we assumed that the flux qubit is operated at the symmetric point.

**Figure 1:**
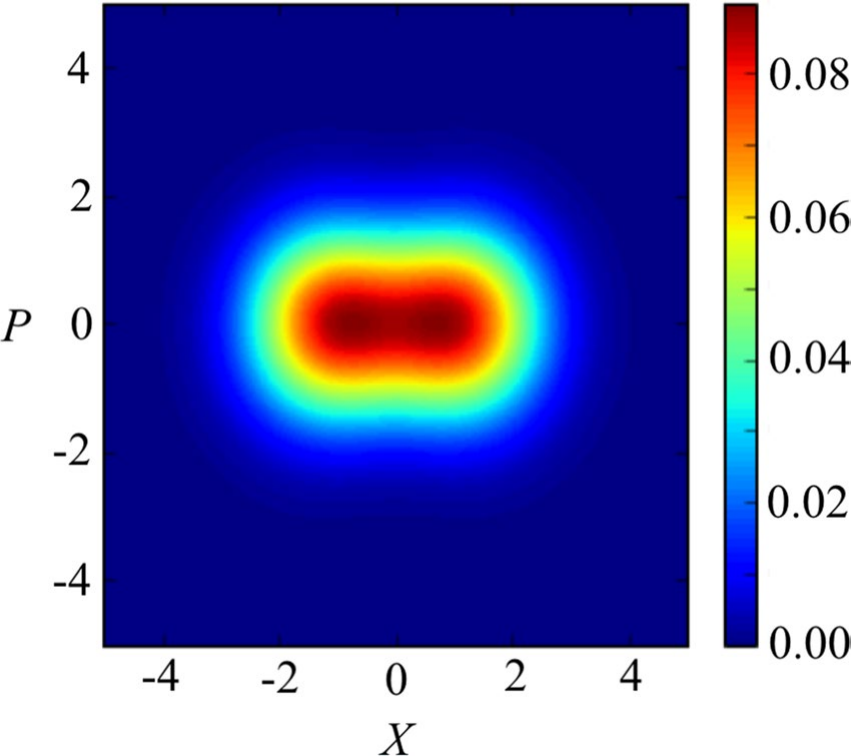
The *Q* function $\langle \beta |\hat{\rho }|\beta \rangle /\pi$ of the reduced density matrix $\hat{\rho }$ of the cavity in the ground state $|G\rangle$, where $\beta =X+iP$. Here ${\omega }_{{\rm{q}}}=2\pi \times 0.299\,{\rm{GHz}}$, $g=2\pi \times 4.920\,{\rm{GHz}}$, ${\omega }_{{\rm{r}}}=2\pi \times 6.336\,{\rm{GHz}}$.

Throughout this paper we assume that the qubit frequency is much smaller than the resonator frequency, allowing us later to use an adiabatic approximation. In this case, in the limit ${\omega }_{{\rm{q}}}\to 0$, we can approximately write the ground state of this system as^17^

$$|G\rangle \simeq \frac{1}{\sqrt{2}}(|R\rangle |\alpha \rangle -|L\rangle |\,-\,\alpha \rangle )$$

where $\alpha =g/{\omega }_{{\rm{r}}}$ is the ratio of the coupling strength $g$ and resonator energy ${\omega }_{{\rm{r}}}$. As an example, using parameters close to those used in^36^, we plot the $Q$ function of the reduced density matrix of $|g\rangle$ where the atom is traced out in Fig. 1. The definition of the $Q$ function for a state $\hat{\rho }$ is $Q(\beta )=\frac{1}{\pi }\langle \beta |\hat{\rho }|\beta \rangle$, where $|\beta \rangle$ is a coherent state for a complex number $\beta$. We plot the real part of $\beta$ in the $x$ axis while we plot the imaginary part of $\beta$ in the $y$ axis.

In this regime, it is straightforward to understand that spectroscopic measurements can excite transitions between eigenstates, and give information on the energy-level structure. However, it is difficult to, for example, probe the relative weights of the $|L\rangle$ and $|R\rangle$ states in the ground-state, or project the entire system into a state which is not in the eigenbasis, because the energy scales of the system are so large compared with the coupling strength between the nonlinear resonator and the flux qubit.

## Using a Nonlinear Resonator As a Measurement Device

Here, as a measurement apparatus, we consider a driven nonlinear resonator whose energy depends on the state of the qubit. It is well understood that a nonlinear resonator can exhibit bistability^50,52,59–61^, which makes such a device sensitive to small changes in external fields. In addition, the nonlinearity induces a rapid change in the photon number under driving^50^, compared to the linear case. When used as a measurement device, the fast evolution and the sensitivity of the steady-state to weak fields result in a strong and fast correlation of the nonlinear resonator state with the qubit being measured, potentially giving a means to implement a rapid projective measurement. One should note that, typically, the state of the nonlinear resonator is itself measured by standard homodyne techniques^58^, and this measurement provides the information about the qubit state.

It is worth mentioning that there are some theoretical proposals^11^ to treat such a measurement device as a two level system when the measurement outcomes are binary. However, since such a simplification cannot easily quantify the strength of the correlation between the target qubit and measurement apparatus during the measurement process, we now model the measurement apparatus with a proper Hamiltonian as we will describe below.

The total system, composed of the ultra-strongly-coupled light-matter system, and the nonlinear resonator measurement device, can be described by the Hamiltonian in the laboratory frame^17,20,21,50,52,60,61^

$$\begin{array}{rcl}{\hat{H}}_{{\rm{tot}}}^{({\rm{lab}})} & = & {\hat{H}}_{{\rm{Rabi}}}+{\hat{H}}_{{\rm{nr}}}^{({\rm{lab}})}+{\hat{H}}_{{\rm{int}}}^{({\rm{lab}})}\\ {\hat{H}}_{{\rm{nr}}}^{({\rm{lab}})} & = & (\delta +{\omega }_{d}){\hat{b}}^{\dagger }\hat{b}-\chi {({\hat{b}}^{\dagger }\hat{b})}^{2}-f\,\cos ({\omega }_{d}t)(\hat{b}+{\hat{b}}^{\dagger })\\ {\hat{H}}_{{\rm{int}}}^{({\rm{lab}})} & = & J{\hat{\sigma }}_{z}{\hat{b}}^{\dagger }\hat{b}\end{array}$$

where $\hat{b}$ is the annihilation operator of the nonlinear system, $\delta$ denotes the detuning between the nonlinear resonator energy and driving frequency, $\chi$ is the nonlinearity strength, $f$ denotes the driving strength of the nonlinear resonator, and ${\omega }_{d}$ is the driving frequency of the nonlinear resonator. In addition, $J$ is the coupling between the qubit and the nonlinear resonator, which is not derived from the dispersive approximation to a dipole coupling, but is intrinsic (see Appendix B for details.). Depending on the state of the qubit, the frequency of the nonlinear resonator changes. We set the parameters of the system such that when the qubit is in $|L\rangle$, the nonlinear resonator experiences the bifurcation effect to generate the high amplitude state. On the other hand, when the state is in $|R\rangle$, the state of the nonlinear resonator stays in the low amplitude state.

It is possible to activate the effective interaction between the measurement apparatus and ultra-strongly coupled system by starting driving the nonlinear resonator, because the vacuum state of the nonlinear resonator without driving makes the effective interaction negligible. In the rotating frame defined by  ${\hat{U}}_{{\rm{rot}}}(t)=\exp [\,-\,i{\omega }_{d}\,t{\hat{b}}^{\dagger }\hat{b}]$, and by applying the rotating-wave approximation, we have

1 $$\begin{array}{rcl}{\hat{H}}_{{\rm{tot}}} & = & {\hat{H}}_{{\rm{Rabi}}}+{\hat{H}}_{{\rm{nr}}}+{\hat{H}}_{{\rm{int}}}\\ {\hat{H}}_{{\rm{nr}}} & = & \delta {\hat{b}}^{\dagger }\hat{b}-\chi {({\hat{b}}^{\dagger }\hat{b})}^{2}-\frac{f}{2}(\hat{b}+{\hat{b}}^{\dagger })\\ {\hat{H}}_{{\rm{int}}} & = & J{\hat{\sigma }}_{z}{\hat{b}}^{\dagger }\hat{b},\end{array}$$

In order to include the loss of photons from the nonlinear resonator, we adopt the following Lindblad master equation, valid when the coupling between nonlinear resonator and its environment is weak, and when the coupling $J$ between the nonlinear resonator and the qubit is weak^50,52,60,61^

2 $$\frac{d}{dt}\hat{\rho }=-\,i[{\hat{H}}_{{\rm{tot}}},\hat{\rho }]+\frac{\kappa }{2}(2b\hat{\rho }{b}^{\dagger }-{\hat{b}}^{\dagger }\hat{b}\hat{\rho }-\hat{\rho }{\hat{b}}^{\dagger }\hat{b}),$$

where $\kappa$ denotes the photon leakage rate from the nonlinear cavity. The potential losses from the ultra-strongly coupled system are described later.

### Coarse-graining of the measurement outcome

**Figure 2:**
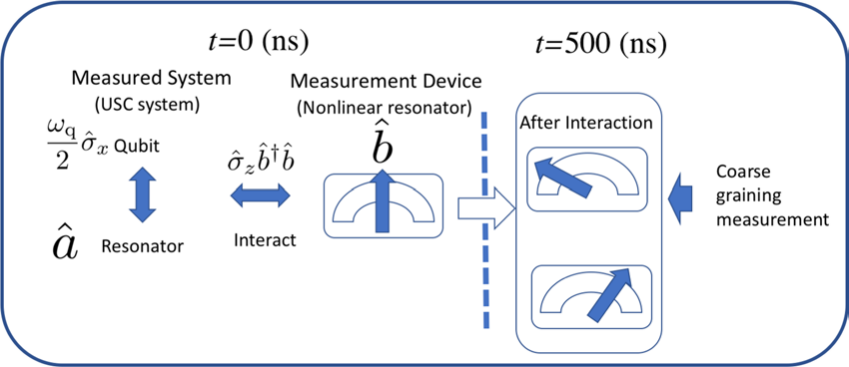
A schematic of the measurement process. The USC system and the nonlinear resonator are allowed to interact for some time *t*, after which the resonator is projected onto two different quadratures. We then analyze the correlation between the conditioned USC system state and the resonator.

After the qubit and the measurement apparatus have been allowed to interact for some time (see Fig. 2), we need to perform a measurement on the measurement apparatus itself. Ideally, one could apply a projection operator ${\hat{P}}_{x}=|x\rangle \langle x|$ on the nonlinear resonator, where $|x\rangle$ is an eigenvector of the quadrature operator $\hat{x}=(\hat{b}+{\hat{b}}^{\dagger })/2$. However, due to imperfections in the measurement setup, one cannot resolve arbitrarily small differences in the state of the resonator. Normally, to describe more realistically the measurement process, one takes this into account by considering the integrated signal-to-noise^62^, where the noise can include contributions from vacuum fluctuations and noise in the measurement apparatus itself. Here, instead we employ a “coarse graining” approximation described by the following operator with Gaussian noise

$${\hat{E}}_{x}=\frac{1}{{\pi }^{1/4}\sqrt{2\sigma }}\,{\int }_{-{\rm{\infty }}}^{{\rm{\infty }}}\,dx{\rm{^{\prime} }}\exp {\textstyle [}-\frac{{(x{\rm{^{\prime} }}-x)}^{2}}{4{\sigma }^{2}}{\textstyle ]}|x{\rm{^{\prime} }}\rangle \langle x{\rm{^{\prime} }}|,$$

where $\sigma$ is the width of the error of the measurement process, and the post-measurement state is described by ${\hat{E}}_{x}\hat{\rho }{\hat{E}}_{x}/{\rm{Tr}}[{\hat{E}}_{x}\hat{\rho }{\hat{E}}_{x}]$. Similar coarse graining approaches have been made in refs. ^63,64^. This approach allows us to consider the transition from small to large noise situations without being specific about the source of the noise.

Correlations between the nonlinear resonator and the qubit should occur after they have interacted for some time, and, for the parameter regime we use in this work, typically the nonlinear resonator state with $x\ge 0$ ($x < 0$) corresponds to an outcome where the qubit was initially in its excited (ground) state. We can describe the post-measurement state of the ultra-strongly-coupled (USC) system as (see Appendix C for details)

$$\begin{array}{c}{\hat{\rho }}_{x\ge 0}=\frac{1}{N}\,{\int }_{-{\rm{\infty }}}^{{\rm{\infty }}}\,dx\,{\rm{e}}{\rm{r}}{\rm{f}}{\rm{c}}{\textstyle (}\,-\,\frac{x}{\sqrt{2}\sigma }{\textstyle )}\langle x|\hat{\rho }|x\rangle \\ {\hat{\rho }}_{x < 0}=\frac{1}{N{\rm{^{\prime} }}}\,{\int }_{-{\rm{\infty }}}^{{\rm{\infty }}}\,dx\,{\rm{e}}{\rm{r}}{\rm{f}}{\rm{c}}\,{\textstyle (}\frac{x}{\sqrt{2}\sigma }{\textstyle )}\langle x|\hat{\rho }|x\rangle ,\end{array}$$

where erfc is the complementary error function and $N$ and *N*′ are normalization factors.

In the limit when $\sigma \to +\,\infty$, we obtain ${\hat{\rho }}_{x\ge 0}={\hat{\rho }}_{x < 0}\propto {\int }_{-\infty }^{\infty }\,dx\langle x|\hat{\rho }|x\rangle$. In this case, the measurement results do not contain any information of the post-measurement state of the qubit. On the other hand, we obtain

$${\hat{\rho }}_{x\ge 0}\propto {\int }_{0}^{\infty }\,dx\langle x|\hat{\rho }|x\rangle ,$$

and

$${\hat{\rho }}_{x < 0}\propto {\int }_{-\infty }^{0}\,dx\langle x|\hat{\rho }|x\rangle ,$$

in the limit $\sigma \to 0$, which corresponds to an ideal projective measurement that can perfectly distinguish $x\ge 0$ or $x < 0$.

## Full Dynamics of the USC System and Nonlinear Measurement Device

Using parameters from^36^, we numerically^65,66^ solve Eq. (2), with the following measurement protocol (see Fig. 2): We assume the USC system is in the initial state $|G\rangle$, and that the nonlinear measurement apparatus is in its uncoupled and undriven ground state. We then allow them (the USC system and the measurement apparatus) to interact for a time up to $t=500\,{\rm{ns}}$. We then apply an instantaneous coarse-grained measurement to the measurement system.

**Figure 3:**
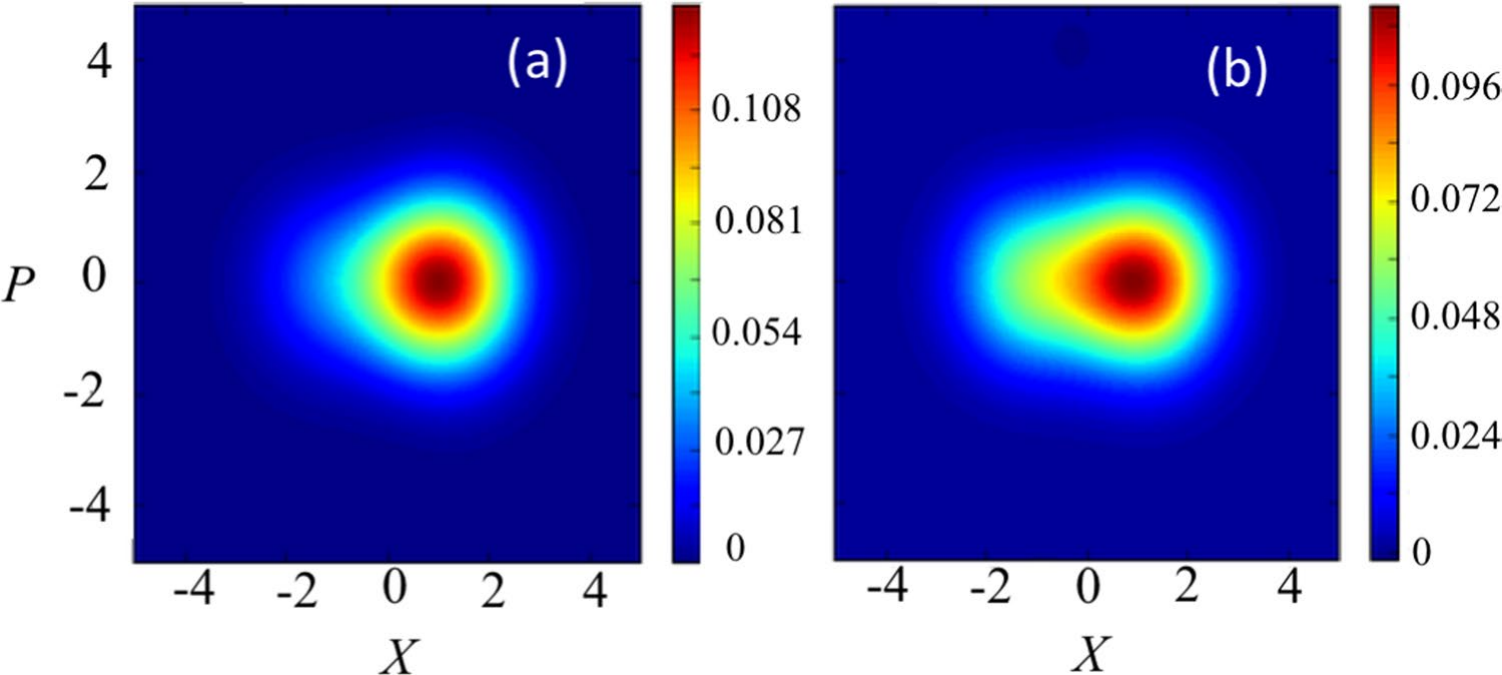
The *Q* function of the reduced density matrix of the resonator ultra-strongly-coupled to the qubit after the coarse graining-measurement. We consider in (**a**) a projection into $x < 0$ at a time $t=500\,{\rm{ns}}$ for $\sigma =0.5$, and (**b**) the same projection for $\sigma =50$. These examples confirm that, as we increase the value of $\sigma$, the change of the $Q$ function induced by the measurement becomes smaller. We set $t=500\,{\rm{ns}}$, ${\omega }_{{\rm{q}}}=2\pi \times 0.299\,{\rm{GHz}}$, $g=2\pi \times 4.920\,{\rm{GHz}}$, ${\omega }_{{\rm{r}}}=2\pi \times 6.336\,{\rm{GHz}}$, $\kappa =2\pi \times 2.375\,{\rm{MHz}}$, $\delta =2\pi \times 5.698\,{\rm{MHz}}$, $\chi =2\pi \times 80.735\,{\rm{kHz}}$, $f=2\pi \times 22.792\,{\rm{MHz}}$, and $J=2\pi \times 949.8\,{\rm{kHz}}$.

To begin, in Fig. 3, we show how the $Q$ function of the resonator part of the USC system depends on the resolution of the coarse-graining measurement. One sees that the change in the state of the USC resonator is much stronger when the measurement resolution is higher (corresponding to a decrease of the coarse-graining variance $\sigma$).

**Figure 4:**
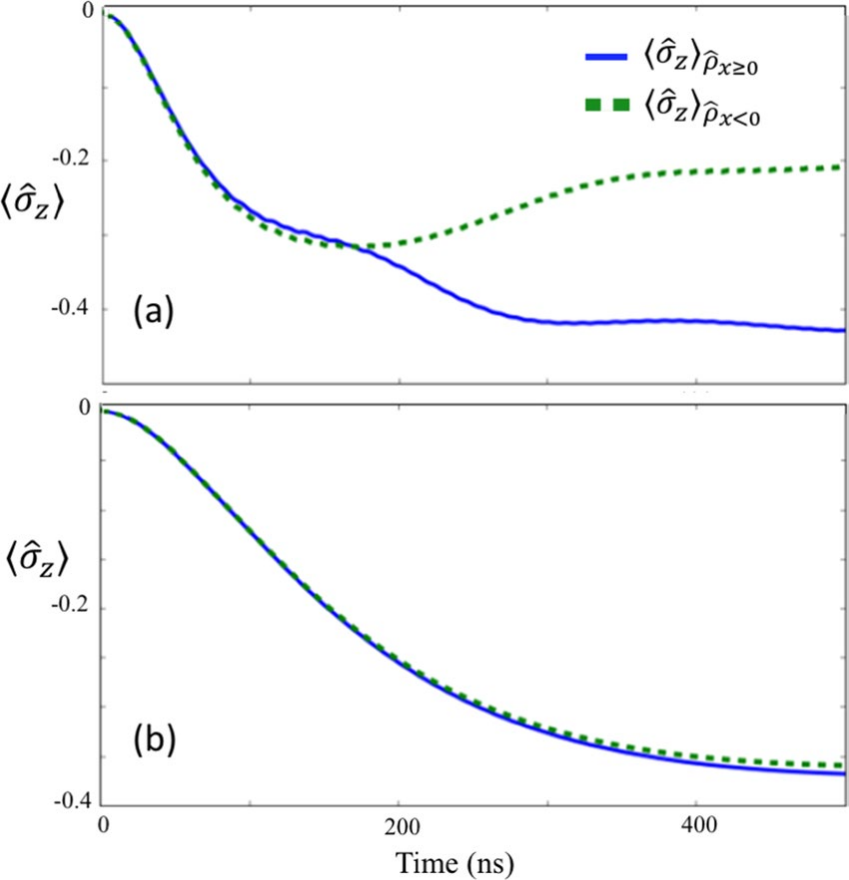
$\langle {\hat{\sigma }}_{z}\rangle$ after the coarse-graining measurements that projects the state into ${\hat{\rho }}_{x\ge 0}$ or ${\hat{\rho }}_{x < 0}$ depending on the measurement results. We plot $\langle {\hat{\sigma }}_{z}\rangle$ for the nonlinear resonator (**a**) and for the linear resonator (**b**) as the measurement apparatus. Here, we set the coarse-graining value as $\sigma =5$. For the other parameters, we use the same as those in Fig. 3.

In addition, in Fig. 4 we plot the post-measurement observable ${\hat{\sigma }}_{z}=|L\rangle \langle L|-|R\rangle \langle R|$ for the state of the qubit, for the different post-measurement outcomes ${\hat{\rho }}_{x\ge 0}$ and ${\hat{\rho }}_{x < 0}$.

**Figure 5:**
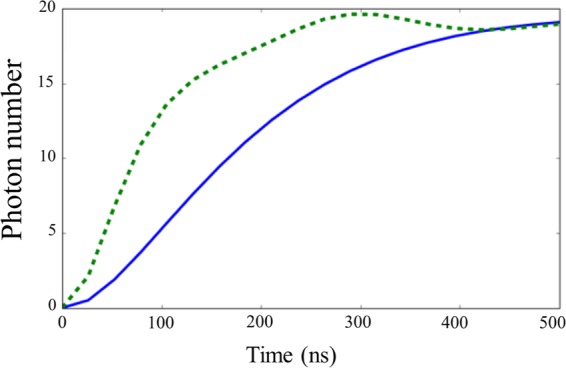
The average number of photons in the nonlinear resonator (dashed green curve) and in the linear resonator (blue continous curve). The parameters used are the same as those in Fig. 4.

For comparison, we consider both a linear resonator ($\chi =0$) and a nonlinear resonator ($\chi \ne 0$) as the measurement devices. In addition, in Fig. 5, we show the average photon number inside the measurement resonator, for the case of a nonlinear and a linear measurement device. We can see that the number of photons at time $t=500$ ns is almost the same. In all figures, for the nonlinear measurement resonator, when we set the coarse-graining value as $\sigma =5$, the post-measurement states of the USC cavity and the qubit change significantly, depending on the measurement outcome, and so we observe a clear measurement backaction on the ultra-strongly coupled system.

The state of the qubit (Fig. 4) depends strongly on the outcome of the coarse-grained measurement in the case of the nonlinear resonator, indicating a clear measurement backaction on the ultra-strongly coupled system. On the other hand, we cannot observe the measurement backaction in the case of a linear resonator. Interestingly, although we set the coupling strength $J$ as approximately 300 times smaller than the qubit energy in these examples, the nonlinear resonator can still affect the state of the system on reasonable time scales.

An ideal result would return perfect post-selection correlations, such that ${\langle {\sigma }_{z}\rangle }_{{\rho }_{x\ge =0}}=-\,1$ and ${\langle {\sigma }_{z}\rangle }_{{\rho }_{x < 0}}=+\,1$, indicating the fidelity of our result is imperfect. However, a naive application of the rotating-wave approximation to the system and measurement device coupling term suggests that the influence of system and measurement apparatus on each other should be entirely negligible, and that there should be no correlation at all.

It is worth mentioning that such an approximation should also take into account the norm of the operator in the interaction term, which, for the driven nonlinear resonator, can be large. In our case, the number of photons in the nonlinear resonator is around 20. Surprisingly, even if we consider the norm of the operator in the interaction term (which corresponds to the number of the photons in the nonlinear resonator), the effective coupling strength ($J$ multiplied by the norm) is still approximately 15 times smaller than the qubit energy in these non-energy eigenbasis measurements. So we can conclude that a clear difference between the case with a finite $J$ and the case without $J$ in our numerical simulations cannot be explained by a simple application of the rotating wave approximation.

In addition, a comparison to a linear measurement device for the same regime shows almost no correlation at all, so while the nonlinear device cannot perfectly project onto eigenstates of ${\sigma }_{z}$, it is capable of performing partial projection. In the next sections we will show that a better result can of course be reached by decreasing ${\omega }_{q}$ or increasing $J$, and we will explain why the nonlinear resonator works, albeit imperfectly, in the difficult parameter regime when $J$ is much smaller than ${\omega }_{q}$.

### Comparison to the QND limit

**Figure 6:**
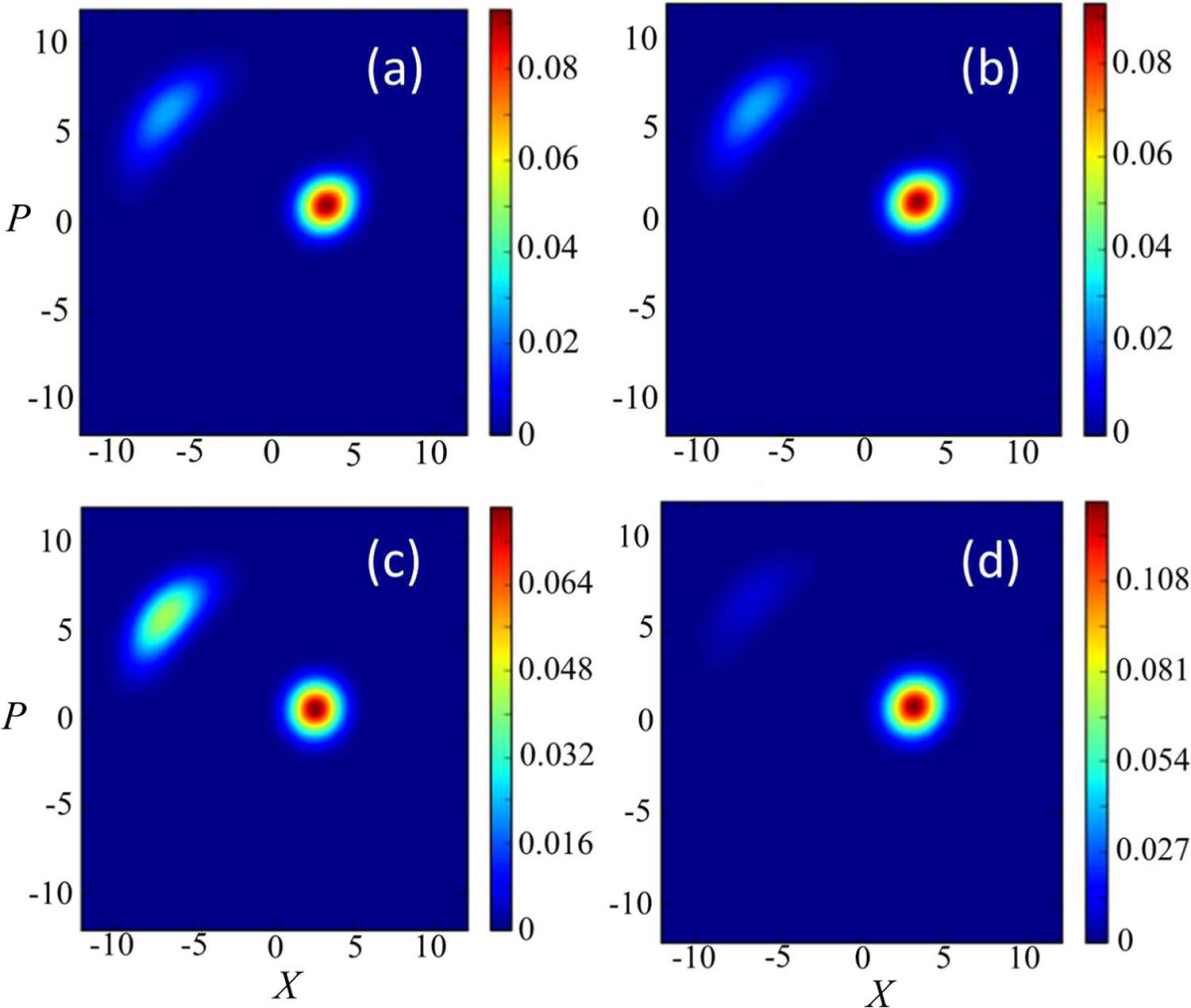
The *Q* functions of the nonlinear resonator for several conditions: (**a**) Numerical simulation of the full Hamiltonian described in Eq. (1). (**b**) The two-level system approximation described by the Hamiltonian in Eq. (3). (**c**) Ideal QND measurement, which is possible in the limit ${\omega }_{{\rm{q}}}=0$. (**d**) When the nonlinear resonator does not couple at all with the qubit. We use the same parameters as those in Fig. 4.

To further compare our non-energy eigenbasis measurements with an ideal quantum non-demolition (QND) measurement, it is instructive to look at the behavior of the $Q$ function of the nonlinear resonator, as shown in Fig. 6. Here, we consider our non-energy eigenbasis measurement, quantum non-demoliton measurements for the limit ${\omega }_{{\rm{q}}}=0$ (which makes the measurement satisfy the QND condition $[{\hat{H}}_{{\rm{Rabi}}},{\hat{H}}_{{\rm{int}}}]=0$), and null measurements with $J=0$.

We observe a clear difference between our non-energy eigenbasis measurements and measurements in the QND limit. In particular, the probability to obtain the high-amplitude state in the nonlinear resonator becomes much larger for QND measurements than that for the non-energy eigenbasis measurement case.

Also, the figures show that, roughly speaking, the probability to obtain the high-amplitude state of the resonator for the non-energy eigenbasis measurements lies between the case of the QND measurements and null measurements.

### Low-energy two-level approximation

To give an intuitive explanation for why the nonlinear resonator measurement apparatus can become strongly correlated with the USC system, even when the coupling between measurement apparatus and system is much smaller than the system energy scales, we introduce a two-level approximation for the USC system. (see Appendix D for details, and a careful analysis of the validity of this approximation). In our simulations, the initial state is $|G\rangle$, and the interaction Hamiltonian $J{\hat{\sigma }}_{z}{\hat{b}}^{\dagger }\hat{b}$ mainly induces a transition from $|G\rangle$ to the first excited state $|E\rangle =\frac{1}{\sqrt{2}}(|R\rangle |\alpha \rangle +|L\rangle |\,-\,\alpha \rangle )$. Since the transition matrix elements of the interaction Hamiltonian to the other excited states are negligible, we can approximate the low-energy states of the ultra-strongly-coupled system as a two-level system. In this case, ${\hat{H}}_{{\rm{Rabi}}}$ and ${\hat{H}}_{{\rm{int}}}$ can be written as

$$\begin{array}{rcl}{\hat{H}}_{{\rm{Rabi}}} & \approx  & \frac{{\omega }_{{\rm{eff}}}}{2}{\hat{\sigma }^{\prime} }_{z}\\ {\hat{H}}_{{\rm{int}}} & \approx  & J{\hat{\sigma }^{\prime} }_{x}{\hat{b}}^{\dagger }\hat{b},\end{array}$$

where

$${\omega }_{{\rm{eff}}}={\omega }_{{\rm{q}}}\,\exp [\,-\,2{\alpha }^{2}],$$

and

$$\begin{array}{rcl}{\hat{\sigma }^{\prime} }_{z} & = & |E\rangle \langle E|-|G\rangle \langle G|\\ {\hat{\sigma }^{\prime} }_{x} & = & |G\rangle \langle E|+|E\rangle \langle G|.\end{array}$$

**Figure 7:**
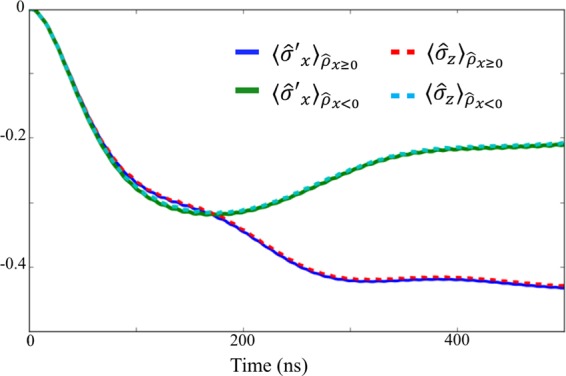
Numerical simulations of the expected values of ${\hat{\sigma }^{\prime} }_{x}$ and ${\hat{\sigma }}_{z}$ after the state is projected into ${\hat{\rho }}_{x\ge 0}$ or ${\hat{\rho }}_{x < 0}$ depending on the measurement results. We use the same parameters as those in Fig. 4.

In Fig. 7, we plot $\langle {\hat{\sigma }^{\prime} }_{x}\rangle$, corresponding to ${\hat{\rho }}_{x\ge 0}$ and ${\hat{\rho }}_{x < 0}$, with this two-level system approximation. To check the validity of this simplified model, we plot $\langle {\hat{\sigma }^{\prime} }_{x}\rangle$ with this model and $\langle {\hat{\sigma }}_{z}\rangle$ using the full model in Fig. 7. These results show an excellent agreement. Also, we confirm that the behavior of the $Q$ function of the nonlinear resonator for the two-level approximation (b) agrees well with the full Hamiltonian case (a), as shown in Fig. 6.

### The role of the AC Stark shift to induce correlations

With this two-level approximation, we can show that the large correlation between the nonlinear resonator and the ultra-strongly-coupled system originates from the combination of an AC Stark shift and an adiabatic transition. It is easy to see that the large number of photons in the nonlinear resonator induces an energy shift (AC Stark shift) of the USC two-level system. Since the photon number of the high-amplitude state is different from that of the low-amplitude state, the size of the AC Stark shift strongly depends on the state of the nonlinear resonator. As long as the timescale of the change in the nonlinear resonator photons is much smaller than $1/{\omega }_{{\rm{eff}}}$, the state of the two-level system remains in the ground state of the following effective Hamiltonian

3 $${\hat{H}}_{{\rm{eff}}}=J{\langle {\hat{b}}^{\dagger }\hat{b}\rangle }_{H(L)}{\hat{\sigma }^{\prime} }_{x}+\frac{{\omega }_{{\rm{eff}}}}{2}{\hat{\sigma }^{\prime} }_{z},$$

where ${\langle {\hat{b}}^{\dagger }\hat{b}\rangle }_{H}$ (${\langle {\hat{b}}^{\dagger }\hat{b}\rangle }_{L}$) is the average photon number of the high (low) amplitude state.

**Figure 8:**
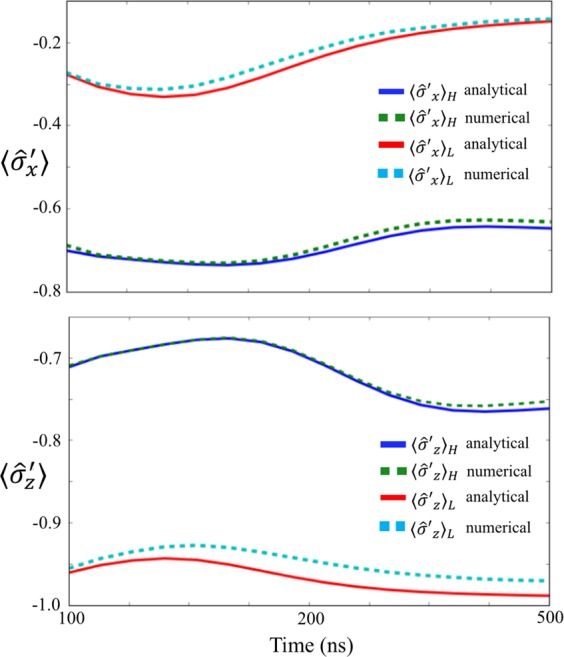
Numerical results and analytical solutions of the expected values of ${\hat{\sigma }^{\prime} }_{x}$ and ${\hat{\sigma }^{\prime} }_{z}$ after the nonlinear resonator is projected into a high-amplitude state or a low-amplitude state. In the analytical calculations, we use the simplified Hamiltonian described in Eq. (3). Note that we do not show the time evolution from $t=0\,{\rm{ns}}$ to $t=100\,{\rm{ns}}$, because the high-amplitude state is not generated until approximately $t=100\,{\rm{ns}}$.

When the nonlinear measurement resonator becomes a mixed state of the low- and high-amplitude states, we expect that the AC Stark shift (whose amplitude depends on the nonlinear resonator state) induces an adiabatic change of the ground state of the two-level system. This leads to a large correlation between the USC system and the measurement resonator. To show the validity of this interpretation, we analytically calculate the $\langle {\hat{\sigma }^{\prime} }_{z(x)}\rangle$ of the ground state of the Hamiltonian in Eq. (3) where we substitute the numerically calculated photon numbers of the high (low) amplitude state for ${\langle {\hat{b}}^{\dagger }\hat{b}\rangle }_{{\rm{H}}}$ (${\langle {\hat{b}}^{\dagger }\hat{b}\rangle }_{{\rm{L}}}$). In Fig. 8, we compare these results with the numerical simulations^65,66^ where the master equation with the simplified Hamiltonian is solved. We plot the result from $t=100\,{\rm{ns}}$ to $t=500\,{\rm{ns}}$, because from $t=0\,{\rm{ns}}$ to $t=100\,{\rm{ns}}$, the high amplitude state is not generated. There is a good agreement between these two results, leading us to conclude that the correlation between the two-level system and the nonlinear resonator is induced by the aforementioned adiabatic changes due to the AC Stark shift, whose amplitude depends on the nonlinear resonator state. For a more detailed explanation of the AC Stark shift, please refer to Appendix F. Also, to quantify such a correlation, in Appendix G, we discuss the evolution of the quantum discord, which also shows a strong correlation even when the coupling strength is much smaller than the system energy scale.

**Figure 9:**
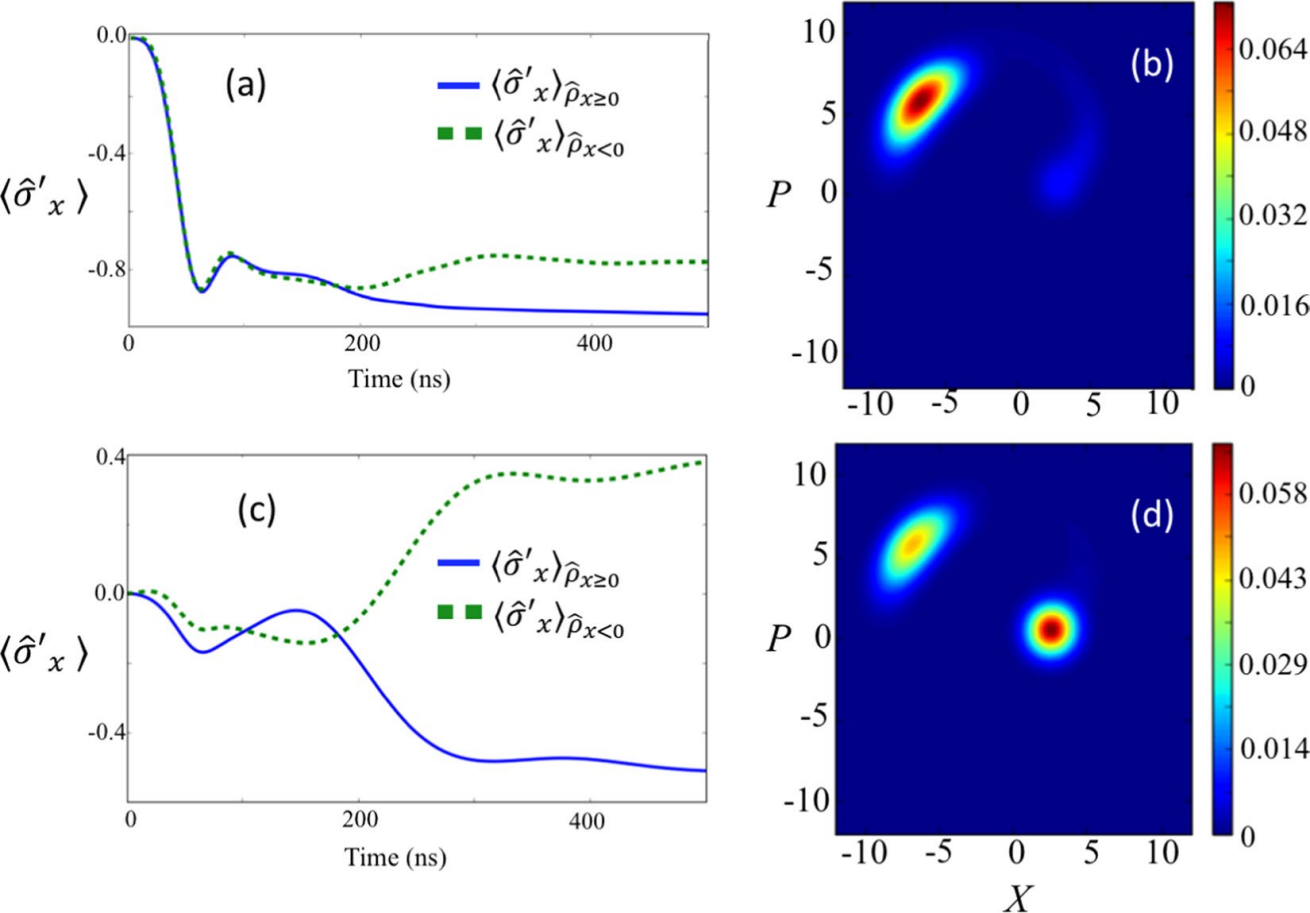
(**a**,**b**) Dynamics of the ultra-strongly-coupled system and the nonlinear resonator when the effective energy ${\omega }_{{\rm{eff}}}$ is 10 times smaller than those in Fig. 4. (**a**) The expected value of ${\hat{\sigma }^{\prime} }_{x}$ after the nonlinear resonator is projected into a high-amplitude state or a low-amplitude state. (**b**) The *Q* function of the nonlinear resonator at time $500\,{\rm{ns}}$. (**c**,**d**) Dynamics of the ultra-strongly-coupled system and the nonlinear resonator when the effective energy ${\omega }_{{\rm{eff}}}$ is 100 times smaller than those in Fig. 4. (**c**) The expected value of ${\hat{\sigma }^{\prime} }_{x}$ after the nonlinear resonator is projected into a high-amplitude state or a low-amplitude state. (**d**) The $Q$ function of the nonlinear resonator at time $500\,{\rm{ns}}$. Except for the effective energy of the ultra-strongly coupled system, we use the same parameters as those in Fig. 4.

Moreover, we increase the ratio $J/{\omega }_{{\rm{q}}}$ to check how the effect of the AC Stark shift will change. In Fig. 9(a), we plot ${\langle {\hat{\sigma }^{\prime} }_{x}\rangle }_{{\hat{\rho }}_{x\ge 0}}$, ${\langle {\hat{\sigma }^{\prime} }_{x}\rangle }_{{\hat{\rho }}_{x < 0}}$, and the $Q$ function at $t=500\,{\rm{ns}}$ where the effective energy ${\omega }_{{\rm{eff}}}$ is 10% of what was used in Fig. 4. Interestingly, although we increase $J/{\omega }_{{\rm{eff}}}$, the backaction of the measurement shown in Fig. 9(a) becomes smaller than that shown in Fig. 4, which is also attributed to the combination of an AC Stark shift and an adiabatic transition. From Fig. 9(a), the system converges into an eigenstate of ${\hat{\sigma }^{\prime} }_{x}$ after the interaction, regardless of the measurement results of the nonlinear resonator. This can be understood by considering that the AC Stark effect $J{\langle {\hat{b}}^{\dagger }\hat{b}\rangle }_{{\rm{H}}({\rm{L}})}$ becomes much larger than the effective energy ${\omega }_{{\rm{eff}}}$, so that the state of the ultra-strongly-coupled system becomes an eigenstate of ${\hat{\sigma }^{\prime} }_{x}$ for both the high amplitude state and low amplitude state. Furthermore, it is worth mentioning that, from Fig. 9(b), the nonlinear resonator before the measurement almost becomes a high-amplitude state. For an ideal quantum projective measurements on the ground state of the ultra-strongly coupled system, the population in the low-amplitude state should be the same as that of the high-amplitude state, and so this result shows that the effective energy ${\omega }_{{\rm{eff}}}$ is still too large to realize a full projective measurement in the persistent current basis.

We also consider a case when the effective energy ${\omega }_{{\rm{eff}}}$ is 1% of that used in Fig. 4. In that case, ${\langle {\hat{\sigma }^{\prime} }_{x}\rangle }_{{\hat{\rho }}_{x < 0}}$ becomes much larger than ${\langle {\hat{\sigma }^{\prime} }_{x}\rangle }_{{\hat{\rho }}_{x\ge 0}}$, and this cannot be explained just by the AC Stark shift. Moreover, from Fig. 9(d), the population of the high-amplitude state becomes comparable with that of the low-amplitude state. Therefore, in this regime, a strong projection of the ground state of the ultra-strongly-coupled system in the non-energy eigenbasis seems to be realized, which can be quantified by calculating the entanglement between the system and measurement apparatus, as shown in the next section.

## Negativity

As a criteria of entanglement, and to understand how correlations between a nonlinear resonator and a USC system develop, we now consider the negativity. Suppose there is a Hilbert space of two systems, ${{\mathscr {H}}}_{A}\,\otimes \,{{\mathscr {H}}}_{B}$ with a state ${\hat{\rho }}_{AB}$. The definition of negativity is

$$N(\hat{\rho })=\frac{\parallel {\hat{\rho }}^{{T}_{A}}\parallel -1}{2}$$

here, ${\hat{\rho }}^{{T}_{A}}$ is the partial transpose of the state ${\hat{\rho }}_{AB}$ taken over a subsystem *A*, and $\Vert \hat{X}\Vert ={\rm{Tr}}\sqrt{{\hat{X}}^{\dagger }\hat{X}}$ is the trace norm^67^. In our case, the subsystem *A* corresponds to the two-level system approximation of the USC system, and *B* to the nonlinear resonator.

**Figure 10:**
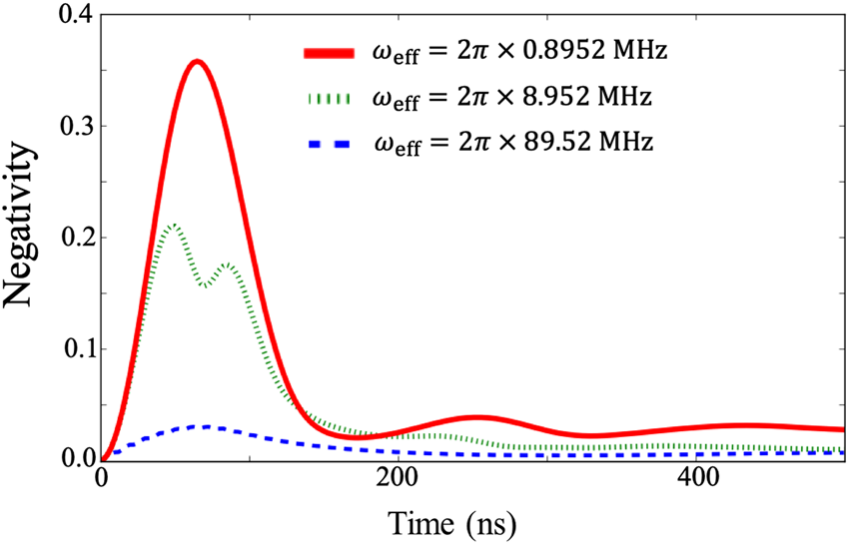
Entanglement between the ultra-strongly coupled system and the nonlinear resonator. We use the negativity as a measure of entanglement. From the top, we plot results with effective energies ${\omega }_{{\rm{eff}}}=2\pi \times 0.8952\,{\rm{MHz}}$, ${\omega }_{{\rm{eff}}}=2\pi \times 8.952\,{\rm{MHz}}$, and ${\omega }_{{\rm{eff}}}=2\pi \times 89.52\,{\rm{MHz}}$. Except for the effective energy of the ultra-strongly coupled system, here we use the same parameters as those in Fig. 4.

In Fig. 10 we plot the negativity to quantify the entanglement between the ultra-strongly-coupled system and the nonlinear resonator. As we increase the ratio $J/{\omega }_{{\rm{q}}}$, the negativity also increases. These results show that a reasonably large entanglement between the ultra-strongly-coupled system and the nonlinear resonator is generated in the regime where we realize a projective measurement on the non-energy eigenbasis. However, due to the decoherence of the nonlinear resonator, the entanglement quickly degrades, and a classical correlation remains in these systems just before the measurement on the nonlinear resonator.

## Conclusions

In conclusion, we investigated non-energy-eigenbasis measurements using a nonlinear resonator measurement apparatus, for the purpose of manipulating an ultra-strong-coupling light-matter system. Here we considered a specific example of a circuit QED system, but the results give us intuitive insights into how non-energy-eigenbasis measurements work in more general scenarios. Interestingly, we found that, even if the effective coupling strength with the measurement device is one order of magnitude smaller than the typical energy scale of the ultra-strongly-coupled system, we can still observe a strong correlation with the measurement device. While this correlation is imperfect, it appears in a difficult parameter regime where traditional linear measurement devices fail completely, and thus maybe practically useful for partial measurement and control of USC systems.

Also, we confirmed that, as one intuitively expects, by increasing the coupling strength with the measurement device, strong entanglement between the system and measurement device can be generated, and we can realize strong projective measurements on the ground state of the ultra-strongly-coupled system.

Nonlinear measurement devices are attractive for their fast and strong response to the system, but they are also difficult to analyze theoretically because of their nonlinear nature. Our results help illuminate the mechanism of how an ultra-strongly coupled system interacts with a nonlinear measurement device in a non-trivial parameter regime, where QND condition is not satisfied

## Appendix

### A. Non-energy-eigenbasis measurements

Here we explain the reason why the non-energy eigenbasis measurement is difficult to realize. Naive calculations indicate that the non-energy eigenbasis measurements would require a violation of the rotating wave approximation, which needs a strong coupling between the system and apparatus. This seems to suggest that, unless the coupling between the system and measurement apparatus is as large as the resonant frequency of the system and measurement apparatus, it would be difficult to implement the non-energy basis measurements. However, our results show that this naive picture is actually wrong if we use the nonlinear resonator as a measurement apparatus.

We can explain these points more quantitatively as follows. Suppose the Hamiltonian which expresses the coupling between a qubit and a linear resonator is as follows,

$$\hat{H}=\frac{{\omega }_{{\rm{eff}}}}{2}{\hat{\sigma }^{\prime} }_{z}+J{\hat{\sigma }^{\prime} }_{x}{\hat{b}}^{\dagger }\hat{b}+{\omega }_{{\rm{r}}}{\hat{b}}^{\dagger }\hat{b}.$$

In the rotating frame defined by a unitary operator  $\hat{U}=\exp [\,-\,i({\omega }_{{\rm{eff}}}{\hat{\sigma }}_{z}/2+{\omega }_{{\rm{r}}}{\hat{b}}^{\dagger }\hat{b})t]$, we obtain

$$\hat{H}(t)=J(\exp [i{\omega }_{{\rm{eff}}}t]{\hat{\sigma }}_{+}^{^{\prime} }+\exp [\,-\,i{\omega }_{{\rm{eff}}}t]{\hat{\sigma }}_{-}^{^{\prime} }){\hat{b}}^{\dagger }\hat{b}.$$

In the limit of a large ${\omega }_{{\rm{eff}}}$, we can use a rotating wave approximation and we obtain

$$\hat{H}(t)\approx 0$$

in the rotating frame.

More generally, we have a Hamiltonian

$$\hat{H}={\hat{H}}_{{\rm{S}}}+J\hat{A}\otimes \hat{B}+{\hat{H}}_{{\rm{E}}},$$

where ${\hat{H}}_{{\rm{S}}}={\sum }_{n}\,{E}_{n}^{({\rm{S}})}|{E}_{n}^{({\rm{S}})}\rangle \langle {E}_{n}^{({\rm{S}})}|$ and ${\hat{H}}_{{\rm{E}}}={\sum }_{m}\,{E}_{m}^{({\rm{E}})}|{E}_{m}^{({\rm{E}})}\rangle \langle {E}_{m}^{({\rm{E}})}|$, (the superindex (S) denotes the system and the superindex (E) denotes the measurement apparatus). In a rotating frame defined by  $\hat{U}=\exp [\,-\,it({\hat{H}}_{{\rm{S}}}+{\hat{H}}_{{\rm{E}}})]$, we have

$$\begin{array}{rcl}\hat{H}(t) & = & J\,\sum _{n,n^{\prime} ,m,m^{\prime} }{C}_{n,n^{\prime} ,m,m^{\prime} }|{E}_{n}^{({\rm{S}})}\rangle \langle {E}_{n^{\prime} }^{({\rm{S}})}|\otimes |{E}_{m}^{({\rm{E}})}\rangle \langle {E}_{m^{\prime} }^{({\rm{E}})}|\\  &  & \times \,\exp [\,-\,i({E}_{n}^{({\rm{S}})}-{E}_{n^{\prime} }^{({\rm{S}})})t-i({E}_{m}^{({\rm{E}})}-{E}_{m^{\prime} }^{({\rm{E}})})t].\end{array}$$

where ${C}_{n,n^{\prime} ,m,m^{\prime} }=\langle {E}_{n}^{({\rm{S}})}|\hat{A}|{E}_{n^{\prime} }^{({\rm{S}})}\rangle \langle {E}_{m}^{({\rm{E}})}|\hat{B}|{E}_{m^{\prime} }^{({\rm{E}})}\rangle$. If the system and measurement apparatus are well detuned, we obtain

$$\hat{H}(t)\approx \sum _{n,m}\,{C}_{n,n,m,m}|{E}_{n}^{({\rm{S}})}\rangle \langle {E}_{n}^{({\rm{S}})}|\otimes |{E}_{m}^{({\rm{E}})}\rangle \langle {E}_{m}^{({\rm{E}})}|,$$

where we used the rotating wave approximation. So the terms that commute with ${\hat{H}}_{{\rm{S}}}$ survive. This clearly shows that we can measure only an observable that commutes with ${\hat{H}}_{{\rm{S}}}$ if the rotating wave approximation is valid. This also means that we need a violation of the rotating wave approximation for the non-energy eigenbasis measurements.

### B. Derivation of the interaction Hamiltonian between the nonlinear resonator and the qubit

In this work we rely on an interaction between a superconducting flux qubit coupled with a frequency tunable resonator. This is not a dispersive approximation to a dipolar coupling. In more detail, the flux qubit is described as

$${\hat{H}}_{{\rm{fq}}}=\frac{\varepsilon }{2}{\hat{\sigma }}_{z}+\frac{\Delta }{2}{\hat{\sigma }}_{x},$$

where $\varepsilon$ denotes the energy bias and Δ denotes the tunneling energy. The Pauli matrix ${\hat{\sigma }}_{z}$ denotes the population of a persistent current basis such as ${\hat{\sigma }}_{z}=|L\rangle \langle L|-|R\rangle \langle R|$, where $|L\rangle$ ($|R\rangle$) denotes a left-sided (right-handed) persistent current.

The frequency tunable resonator is described as

$${\hat{H}}_{r}=\omega (\Phi ){\hat{b}}^{\dagger }\hat{b},$$

where $\omega (\Phi )$ denotes the frequency of the resonator. We assume that the resonator contains a SQUID structure, and we can tune the frequency of the resonator by changing an applied flux penetrating the SQUID structure. (For example, see ref. ^68^).

We can derive the interaction between the flux qubit and the resonator as follows. The persistent current states of the flux qubit induce magnetic fields due to the Biot-Savart law, and this changes the penetrating magnetic flux of the SQUID in the resonator. So the frequency of the resonator depends on the state of the flux qubit. Suppose that $\delta \Phi$ (−$\delta \Phi$) denotes the magnetic flux from the $|L\rangle$ ($|R\rangle$) state, and the resonator frequency will be approximately shifted by $\frac{d\omega }{d\Phi }\delta \Phi$ $(\,-\,\frac{d\omega }{d\Phi }\delta \Phi )$. This provides us with the following Hamiltonian.

$${\hat{H}}_{I}=g|L\rangle \langle L|\otimes {\hat{b}}^{\dagger }\hat{b}-g|R\rangle \langle R|\otimes {\hat{b}}^{\dagger }\hat{b}=g{\hat{\sigma }}_{z}\otimes {\hat{b}}^{\dagger }\hat{b},$$

where $g=\frac{d\omega }{d\Phi }\delta \Phi$. A similar Hamiltonian has been derived in^69^ to represent the coupling between an NV center and a flux qubit. We now assume a large detuning between the flux qubit and resonator. In this case, the dipolar coupling is negligible.

### C. Derivation of the coarse-graining measurement

In the case where there is noise in the measurement apparatus, when we have a position measurement, even if the result of the measurement apparatus is $x$, the real value is not necessarily $x$. To model such situations, we define a measurement operator as follows

$${\hat{E}}_{x}=\frac{1}{{\pi }^{1/4}\sqrt{2\sigma }}\,{\int }_{-{\rm{\infty }}}^{{\rm{\infty }}}\,dx{\rm{^{\prime} }}\,\exp \,{\textstyle [}-\frac{{(x{\rm{^{\prime} }}-x)}^{2}}{4{\sigma }^{2}}{\textstyle ]}|x{\rm{^{\prime} }}\rangle \langle x{\rm{^{\prime} }}|,$$

where $\sigma$ describes the strength of the noise. Also, ${\hat{E}}_{x}$ satisfies the normalization condition

$${\int }_{-\infty }^{\infty }\,{\hat{E}}_{x}^{\dagger }{\hat{E}}_{x}dx=I.$$

Here we consider a composite system which comprises of a system which we hope to readout (ultra-strongly coupled system) and its probe (nonlinear resonator). Also, the measurement result is divided into $x\ge 0$ and $x < 0$. When we have a measurement on a composite system $\hat{\rho }$, the post-measurement state when the result is $x\ge 0$ becomes

$$\begin{array}{ccc}\frac{{\int }_{0}^{{\rm{\infty }}}\,dx{\hat{E}}_{x}\hat{\rho }{\hat{E}}_{x}^{\dagger }}{{\rm{T}}{\rm{r}}[{\int }_{0}^{{\rm{\infty }}}\,dx{\hat{E}}_{x}\hat{\rho }{\hat{E}}_{x}^{\dagger }]} & = & \frac{1}{N}\,{\int }_{0}^{{\rm{\infty }}}\,dx\,{\int }_{-{\rm{\infty }}}^{{\rm{\infty }}}\,d{x}^{{\rm{^{\prime} }}}\,{\int }_{-{\rm{\infty }}}^{{\rm{\infty }}}\,dx{\rm{^{\prime} }}{\rm{^{\prime} }}\exp \,{\textstyle [}-\frac{{({x}^{{\rm{^{\prime} }}}-x)}^{2}}{4{\sigma }^{2}}-\frac{{(x{\rm{^{\prime} }}{\rm{^{\prime} }}-x)}^{2}}{4{\sigma }^{2}}{\textstyle ]}\,\langle {x}^{{\rm{^{\prime} }}}|\hat{\rho }|x{\rm{^{\prime} }}{\rm{^{\prime} }}\rangle |{x}^{{\rm{^{\prime} }}}\rangle \langle x{\rm{^{\prime} }}{\rm{^{\prime} }}|,\end{array}$$

where

$$N={\int }_{0}^{{\rm{\infty }}}\,dx\,{\int }_{-{\rm{\infty }}}^{{\rm{\infty }}}\,dx{\rm{^{\prime} }}{\rm{^{\prime} }}\,\exp \,{\textstyle [}-\frac{{(x{\rm{^{\prime} }}{\rm{^{\prime} }}-x)}^{2}}{2{\sigma }^{2}}{\textstyle ]}\,{\rm{T}}{\rm{r}}[\langle x{\rm{^{\prime} }}{\rm{^{\prime} }}|\hat{\rho }|x{\rm{^{\prime} }}{\rm{^{\prime} }}\rangle ].$$

By tracing out the probe system, we have the post-measurement state of the system ${\hat{\rho }}_{x\ge 0}$ we hope to readout as

$${\hat{\rho }}_{x\ge 0}=\frac{1}{N}\,{\int }_{0}^{{\rm{\infty }}}\,dx\,{\int }_{-{\rm{\infty }}}^{{\rm{\infty }}}\,dx{\rm{^{\prime} }}{\rm{^{\prime} }}\,\exp \,{\textstyle [}\frac{{(x{\rm{^{\prime} }}{\rm{^{\prime} }}-x)}^{2}}{2{\sigma }^{2}}{\textstyle ]}\langle x{\rm{^{\prime} }}{\rm{^{\prime} }}|\hat{\rho }|x{\rm{^{\prime} }}{\rm{^{\prime} }}\rangle .$$

Substituting $t=\frac{x-x^{\prime} }{\sqrt{2}\sigma }$, ${\hat{\rho }}_{x\ge 0}$ can be rewritten as

$${\hat{\rho }}_{x\ge 0}=\frac{1}{N}\,{\int }_{-{\rm{\infty }}}^{{\rm{\infty }}}\,dx\,{\rm{e}}{\rm{r}}{\rm{f}}{\rm{c}}{\textstyle (}-\frac{x}{\sqrt{2}\sigma }{\textstyle )}\langle x|\hat{\rho }|x\rangle ,$$

where ${\rm{erfc}}(x)$ is a complementary error function, and is defined as

$${\rm{erfc}}(x)=\frac{2}{\sqrt{\pi }}\,{\int }_{x}^{\infty }\,\exp (\,-\,{t}^{2})dt.$$

In the limit of $\sigma \to +\,0$, we have

$${\hat{\rho }}_{x\ge 0}=\frac{1}{N}\,{\int }_{0}^{\infty }\,dx\langle x|\hat{\rho }|x\rangle ,$$

which is a noiseless measurement. Also, in the limit of $\sigma \to +\,\infty$, we obtain

$${\hat{\rho }}_{x\ge 0}=\frac{1}{N}\,{\int }_{-\infty }^{\infty }\,dx\langle x|\hat{\rho }|x\rangle ,$$

which shows that we cannot extract any information from the system.

### D. Validity of the two-level approximation

#### D.1 Adiabatic approximation to the Rabi Hamiltonian

We now explain the adiabatic approximation to the Rabi Hamiltonian, that has also been used in previous works^17,19–21^. We will show that, within the framework of the adiabatic approximation, the ultra-strongly coupled system can be treated as a two-level system. The conventional Rabi Hamiltonian can be written as

4 $${\hat{H}}_{{\rm{Rabi}}}=\frac{{\omega }_{{\rm{q}}}}{2}{\hat{\sigma }}_{x}+g(\hat{a}+{\hat{a}}^{\dagger }){\hat{\sigma }}_{z}+{\omega }_{{\rm{r}}}{\hat{a}}^{\dagger }\hat{a}.$$

The adiabatic approximation can be done when ${\omega }_{q}\ll (g,{\omega }_{r})$ and the Rabi Hamiltonian can be diagonalized using the bases

$$|L\rangle |{N}_{-}\rangle =|L\rangle \hat{D}(\,-\,\alpha )|N\rangle ,\,|R\rangle |{N}_{+}\rangle =|R\rangle \hat{D}(\alpha )|N\rangle ,$$

where $\alpha =g/{\omega }_{{\rm{r}}}$, $|L\rangle$ and $|R\rangle$ are eigenstates of ${\hat{\sigma }}_{z}$, $|N\rangle$ is the eigenstates of ${\hat{a}}^{\dagger }\hat{a}$, and $\hat{D}(\alpha )$ is a displacement operator. The states $|L\rangle \hat{D}(\,-\,\alpha )|N\rangle$ and $|R\rangle \hat{D}(\alpha )|N\rangle$ are degenerate in energy and their energy is ${ {\mathcal E} }_{N}={\omega }_{{\rm{r}}}(N-{\alpha }^{2})$. Then, considering that the term ${\omega }_{q}/2{\hat{\sigma }}_{x}$ couples these terms, and only the transitions between the states of the same $N$ are taken into account in the adiabatic approximation, the Rabi hamiltonian can be rewritten as

$$\begin{array}{rcl}{\hat{H}}_{{\rm{Rabi}}} & \approx  & \sum _{N=0}\,[({ {\mathcal E} }_{N}+\frac{{\omega }_{{\rm{q}}}}{2}\langle {N}_{-}|{N}_{+}\rangle )|{\psi }_{N}^{+}\rangle \langle {\psi }_{N}^{+}|\\  &  & +\,({ {\mathcal E} }_{N}-\frac{{\omega }_{{\rm{q}}}}{2}\langle {N}_{-}|{N}_{+}\rangle )|{\psi }_{N}^{-}\rangle \langle {\psi }_{N}^{-}|],\end{array}$$

where

$$|{\psi }_{N}^{\pm }\rangle =\frac{1}{\sqrt{2}}(|L\rangle |{N}_{-}\rangle \pm |R\rangle |{N}_{+}\rangle ),$$

whose eigenvalues are

$${ {\mathcal E} }_{N\pm }={ {\mathcal E} }_{N}\pm \frac{{\omega }_{{\rm{q}}}}{2}\langle {N}_{-}|{N}_{+}\rangle .$$

Also, it can be easily shown that

$$\langle {\psi }_{N}^{\pm }|{\hat{\sigma }}_{z}|{\psi }_{M}^{\pm }\rangle =0,\,\langle {\psi }_{N}^{\mp }|{\hat{\sigma }}_{z}|{\psi }_{M}^{\pm }\rangle ={\delta }_{NM}.$$

So, as long as we apply the adiabatic approximation, the transition due to the ${\hat{\sigma }}_{z}$ term is between $|{\psi }_{N}^{+}\rangle$ and $|{\psi }_{N}^{-}\rangle$. Since the interaction between the ultra-strongly coupled system and the nonlinear resonator can be expressed as $J{\hat{\sigma }}_{z}{\hat{b}}^{\dagger }\hat{b}$, it is possible for us to consider that the ultra-strongly coupled system is driven only by the ${\hat{\sigma }}_{z}$ operator. Also, if the initial state is $|{\psi }_{0}^{-}\rangle$ and the perturbation term is proportional only to ${\hat{\sigma }}_{z}$, the dynamics is limited to $|{\psi }_{0}^{\pm }\rangle$. Therefore, as long as the adiabatic approximation is valid, we can consider our subsystem of the ultra-strongly coupled system as a two-level system.

#### D.2 Estimation of the deviation from the two-level approximation

By calculating the deviation from the two-level approximation, we show a quantitative analysis of how accurate the two-level system approximation is in our parameter regime. We consider a fidelity between the true ground state $|G\rangle$ (the first excited state $|E\rangle$) and $|{\psi }_{0}^{-}\rangle$ ($|{\psi }_{0}^{+}\rangle$). It is possible to estimate the accuracy of our two-level approximation from this fidelity, and we derive a condition of the fidelity to be close to unity. Now, we define

$$\begin{array}{rcl}{\hat{H}}_{0} & = & \sum _{N=0}\,[({ {\mathcal E} }_{N}+\frac{{\omega }_{{\rm{q}}}}{2}\langle {N}_{-}|{N}_{+}\rangle )|{\psi }_{N}^{+}\rangle \langle {\psi }_{N}^{+}|\\  &  & +\,({ {\mathcal E} }_{N}-\frac{{\omega }_{{\rm{q}}}}{2}\langle {N}_{-}|{N}_{+}\rangle )|{\psi }_{N}^{-}\rangle \langle {\psi }_{N}^{-}|]\end{array}$$

and

$$\hat{H}^{\prime} ={\hat{H}}_{{\rm{Rabi}}}-{\hat{H}}_{0}.$$

Here, ${\hat{H}}_{{\rm{Rabi}}}$ is the one defined in Eq. (4). In this way, we regard ${\hat{H}}_{0}$ as the non-perturbative Hamiltonian and $\hat{H}^{\prime}$ the perturbative Hamiltonian. By performing a perturbative calculation up to the lowest order, we obtain

$$|G\rangle \approx \frac{1}{\sqrt{{\mathscr {N}}}}(|{\psi }_{0}^{-}\rangle +|{G}^{(0)}\rangle ),$$

where ${\mathscr {N}}$ is a normalization factor. Then by using perturbation theory, we have

$$\begin{array}{rcl}|{G}^{(0)}\rangle  & = & \sum _{N}\,({c}_{N}^{+}|{\psi }_{N}^{+}\rangle +{c}_{N}^{-}|{\psi }_{N}^{-}\rangle ),\\ {c}_{N}^{+} & = & -\frac{\langle {\psi }_{N}^{+}|\hat{H}^{\prime} |{\psi }_{0}^{-}\rangle }{{ {\mathcal E} }_{N+}-{ {\mathcal E} }_{0-}},\\ {c}_{N}^{-} & = & -\frac{\langle {\psi }_{N}^{-}|\hat{H}^{\prime} |{\psi }_{0}^{-}\rangle }{{ {\mathcal E} }_{N-}-{ {\mathcal E} }_{0-}}.\end{array}$$

It can be easily shown that

$$\langle {\psi }_{N}^{+}|\hat{H}^{\prime} |{\psi }_{0}^{-}\rangle =\langle {\psi }_{N}^{+}|{\hat{H}}_{{\rm{Rabi}}}|{\psi }_{0}^{-}\rangle ={\omega }_{{\rm{q}}}/2\langle {\psi }_{N}^{+}|{\hat{\sigma }}_{x}|{\psi }_{0}^{-}\rangle ,$$

and

$$\langle {\psi }_{N}^{-}|\hat{H}^{\prime} |{\psi }_{0}^{-}\rangle ={\omega }_{{\rm{q}}}/2\langle {\psi }_{N}^{-}|{\hat{\sigma }}_{x}|{\psi }_{0}^{-}\rangle .$$

Then, we have

$$\begin{array}{rcl}|G\rangle  & \approx  & \frac{1}{\sqrt{{\mathscr {N}}}}(|{\psi }_{0}^{-}\rangle +\frac{{\omega }_{{\rm{q}}}}{2}\,\sum _{N=2,4,\mathrm{.}.}\,\tfrac{{(2\alpha )}^{N}}{({ {\mathcal E} }_{N-}+\omega {\alpha }^{2}+{\omega }_{{\rm{q}}}\,/\,2\langle 0-|0+\rangle )\sqrt{N!}}|{\psi }_{N}^{-}\rangle \\  &  & +\,\frac{{\omega }_{{\rm{q}}}}{2}\,\sum _{N=1,3,\mathrm{.}.}\,\tfrac{{(2\alpha )}^{N}}{({ {\mathcal E} }_{N+}+\omega {\alpha }^{2}+{\omega }_{{\rm{q}}}\,/\,2\langle 0-|0+\rangle )\sqrt{N!}}|{\psi }_{N}^{+}\rangle ).\end{array}$$

By assuming ${\omega }_{{\rm{q}}}\ll {\omega }_{{\rm{r}}}$, we have

$$\begin{array}{rcl}{ {\mathcal E} }_{N\pm } & = & {\omega }_{{\rm{r}}}(N-{\alpha }^{2})\pm \frac{{\omega }_{{\rm{q}}}}{2}\langle N\,-\,|N\,+\,\rangle \\  & \approx  & {\omega }_{{\rm{r}}}(N-{\alpha }^{2}),\end{array}$$

and, we obtain

5 $$\begin{array}{rcl}|G\rangle  & \approx  & \frac{1}{\sqrt{{\mathscr {N}}}}[[|{\psi }_{0}^{-}\rangle +\frac{{\omega }_{{\rm{q}}}}{2}{e}^{-2{\alpha }^{2}}(\sum _{N=2,4,\mathrm{.}.}\,\frac{{(2\alpha )}^{N}}{{\omega }_{{\rm{r}}}\,N\sqrt{N!}}|{\psi }_{N}^{-}\rangle \\  &  & +\,\sum _{N=1,3,\mathrm{.}.}\,\frac{{(2\alpha )}^{N}}{{\omega }_{{\rm{r}}}\,N\sqrt{N!}}|{\psi }_{N}^{+}\rangle )],\end{array}$$

where

$${\mathscr {N}}=1+\frac{{\omega }_{{\rm{q}}}^{2}}{{\omega }_{{\rm{r}}}^{2}}{e}^{-4{\alpha }^{2}}\,\mathop{\sum }\limits_{N=1}^{\infty }\,\frac{{(4{\alpha }^{2})}^{N}}{{N}^{2}N!}.$$

Also, we set $\frac{{\omega }_{{\rm{q}}}}{2}\langle 0\,-\,|0\,+\,\rangle \approx 0$.

Similarly, with regard to the first excited state, we can obtain

6 $$\begin{array}{rcl}|E\rangle  & \approx  & \frac{1}{\sqrt{{\mathscr {N}}}}[|{\psi }_{0}^{+}\rangle -\frac{{\omega }_{{\rm{q}}}}{2}{e}^{-2{\alpha }^{2}}(\sum _{N=2,4,\mathrm{.}.}\,\frac{{(2\alpha )}^{N}}{{\omega }_{{\rm{r}}}\,N\sqrt{N!}}|{\psi }_{N}^{+}\rangle \\  &  & +\,\sum _{N=1,3,\mathrm{.}.}\,\frac{{(2\alpha )}^{N}}{{\omega }_{{\rm{r}}}\,N\sqrt{N!}}|{\psi }_{N}^{-}\rangle )].\end{array}$$

(Note that ${\mathscr {N}}$ in Eqs. (5) and (6) are the same.) The fidelity ${F}_{{\rm{G}}}=|\langle {\psi }_{0}^{-}|G\rangle {|}^{2}$ and ${F}_{{\rm{E}}}=|\langle {\psi }_{0}^{+}|E\rangle {|}^{2}$ are calculated as

$${F}_{G}={F}_{E}=\frac{1}{{\mathscr{N}}}={{\textstyle [}1+\frac{{\omega }_{{\rm{q}}}^{2}}{4{\omega }_{{\rm{r}}}^{2}}{e}^{-4{\alpha }^{2}}\mathop{\sum }\limits_{N=1}^{{\rm{\infty }}}\frac{{(4{\alpha }^{2})}^{N}}{{N}^{2}N!}{\textstyle ]}}^{-1}.$$

Then, we define

$$f\equiv \frac{{\omega }_{{\rm{q}}}^{2}}{4{\omega }_{{\rm{r}}}^{2}}\,\exp [\,-\,4{\alpha }^{2}]\,\mathop{\sum }\limits_{N=1}^{\infty }\,\frac{{(4{\alpha }^{2})}^{N}}{{N}^{2}N!}.$$

For $f\ll 1$, we have ${F}_{{\rm{G}}}={F}_{{\rm{E}}}\approx 1-f$, and so we can consider *f* as an infidelity.

**Figure 11:**
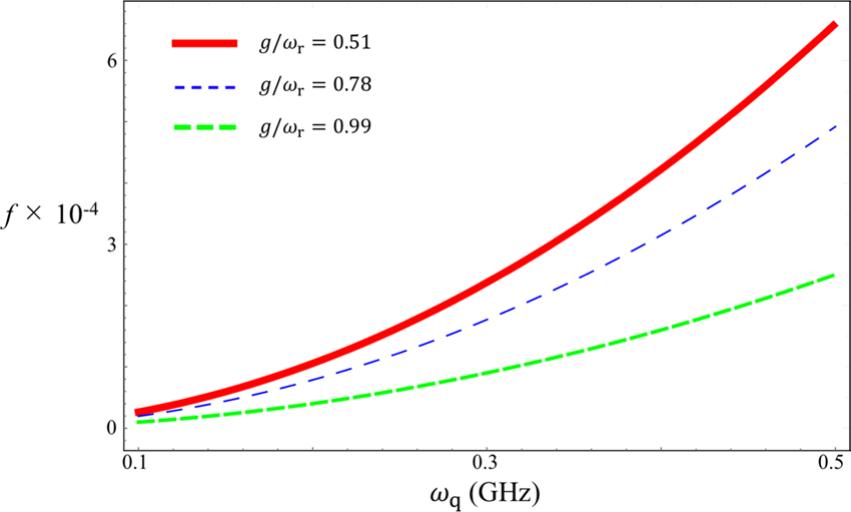
The infidelity $f$ versus qubit frequency ${\omega }_{q}$ for three regimes $g/{\omega }_{{\rm{r}}}=0.51$ (red solid), $g/{\omega }_{{\rm{r}}}=0.78$ (blue dashed), $g/{\omega }_{{\rm{r}}}=0.99$ (green dashed). Here ${\omega }_{{\rm{q}}}$ varies from $2\pi \times 0.1\,{\rm{GHz}}$ to $2\pi \times 0.5\,{\rm{GHz}}$, where ${\omega }_{{\rm{r}}}=2\pi \times 6.336\,{\rm{GHz}}$.

We plot  *f* for three regimes $g/{\omega }_{{\rm{r}}}=0.51,0.78,0.99$. Here, we fix ${\omega }_{{\rm{r}}}=2\pi \times 6.336\,{\rm{GHz}}$. From Fig. 11, we can see that in these regimes the infidelity *f* is sufficiently small.

**Figure 12:**
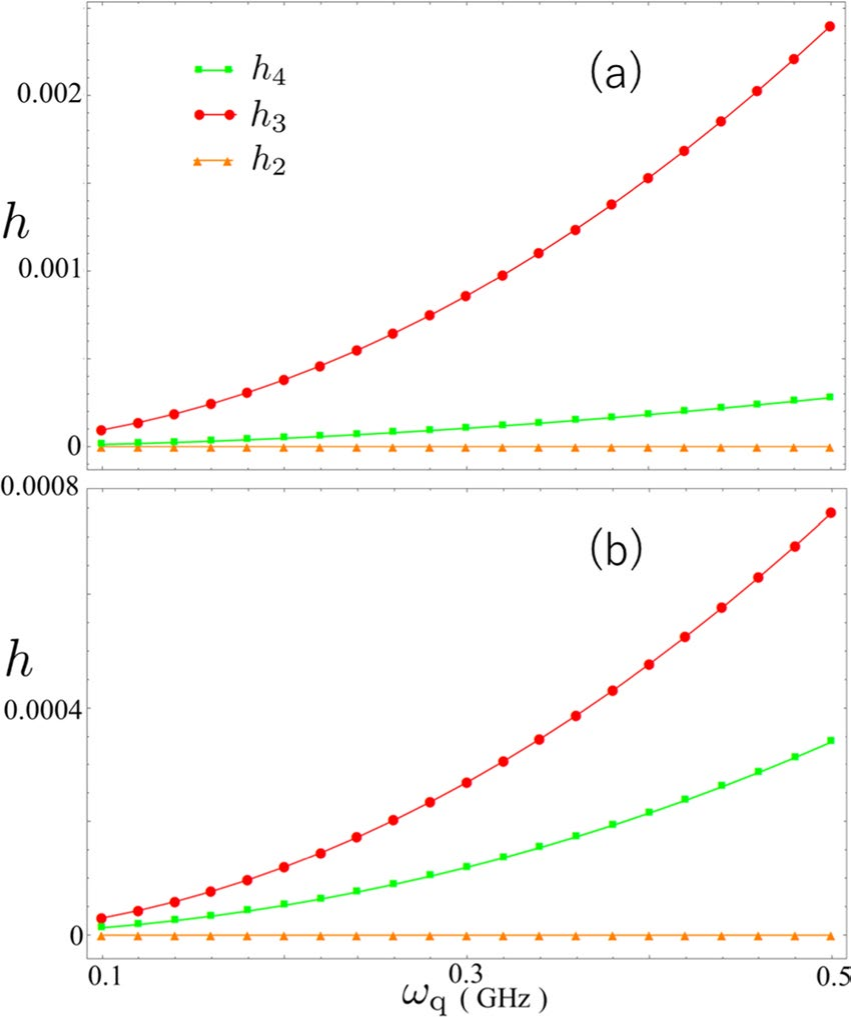
A measure of the leakage to excited states ${h}_{2(3,4)}$ versus qubit frequency ${\omega }_{q}$. Again ${\omega }_{{\rm{q}}}$ varies from $2\pi \times 0.1\,{\rm{GHz}}$ to $2\pi \times 0.5\,{\rm{GHz}}$. Here we set (**a**) $g/{\omega }_{{\rm{r}}}=0.51$, (**b**) $g/{\omega }_{{\rm{r}}}=0.99$, where ${\omega }_{{\rm{r}}}=2\pi \times 6.336\,{\rm{GHz}}$.

Also, we plot the numerically calculated

$${h}_{j}=|\langle {\phi }_{j}|{\hat{\sigma }}_{z}|G\rangle {|}^{2},$$

(*j* = 2, 3, 4) in Fig. 12 in the same regime, where $|{\phi }_{2}\rangle$, $|{\phi }_{3}\rangle$, $|{\phi }_{4}\rangle$ are the second, third and fourth excited states, respectively. This shows the leakage from $|G\rangle$ to unwanted states.

From Figs. 11 and 12, we confirm that, as long as $f\ll 1$ is satisfied, transitions from $|G\rangle$ to the unwanted states such as $|{\phi }_{2}\rangle$, $|{\phi }_{3}\rangle$, $|{\phi }_{4}\rangle$ are small, so that the two-level approximation should be valid in this regime.

### E. Losses in the ultra-strongly coupled system

So far we did not include a full analysis of the USC losses because we assumed that the time scale of such losses would be much longer than the readout time. For completeness, here we present a brief analysis of the influence of such losses. Because including bath-induced transitions between all eigenstates in the full space is complex, here we restrict ourselves to the two-level approximation. We justify this approximation, in our relevant parameter regime, in the previous sections.

The interaction Hamiltonian between our ultra-strongly coupled system and its environment is described by

$${\hat{H}}_{I}=\hat{A}\hat{X}+\hat{B}{\hat{\sigma }}_{z},$$

where $\hat{X}=\hat{a}+{\hat{a}}^{\dagger }$ denotes the position operator and $\hat{A}$ ($\hat{B}$) denotes the environmental operator coupled with the resonator (qubit). Also, we incorporate the effect of a dephasing bath classically modeled as

$${\hat{H}}_{{\rm{dep}}}=f(t){\hat{\sigma }}_{z},$$

where $f(t)$ is a time-dependent random variable and the ensemble average of $f(t)$ is zero. In this case, it is well know that the Born-Markov secular Lindblad master equation can be written in the form^20^

$$\begin{array}{rcl}\frac{d\hat{\rho }}{dt} & = & -i[{\hat{H}}_{s},\hat{\rho }]+\sum _{j,k > j}\,({\Gamma }_{{\sigma }_{z}}^{jk}+{\Gamma }_{X}^{jk}) {\cal{D}} [|j\rangle \langle k|](\hat{\rho })\\  &  & +\,\sum _{j}\, {\cal{D}} [{\Phi }_{j}^{{\rm{dep}}}|j\rangle \langle j|](\hat{\rho })\\  &  & +\,\sum _{j,k\ne j}\,{\Gamma }_{{\rm{dep}}}^{jk} {\cal{D}} [|j\rangle \langle k|](\hat{\rho })\\  {\cal{D}} [\hat{A}](\hat{\rho }) & = & \frac{1}{2}(2\hat{A}\hat{\rho }{\hat{A}}^{\dagger }-{\hat{A}}^{\dagger }\hat{A}\hat{\rho }-\hat{\rho }{\hat{A}}^{\dagger }\hat{A})\end{array}$$

where ${\hat{H}}_{s}$ is the system Hamiltonian and

$${\Gamma }_{{\sigma }_{z}}^{jk}={\kappa }_{q}({\Delta }_{jk})|\langle j|{\hat{\sigma }}_{z}|k\rangle {|}^{2},\,{\Gamma }_{X}^{jk}={\kappa }_{r}({\Delta }_{jk})|\langle j|\hat{X}|k\rangle {|}^{2}.$$

**Figure 13:**
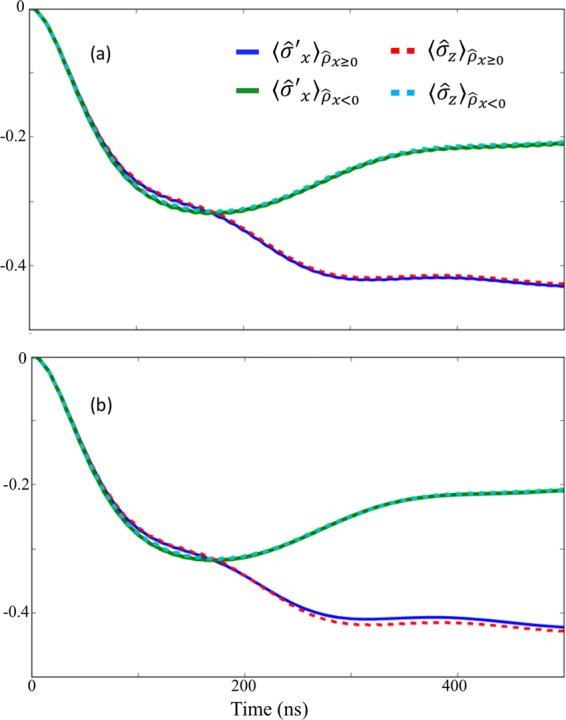
The expectation values $\langle {\hat{\sigma }}_{z}\rangle$ (using the full Hamiltonian in Eq. (1)) and $\langle {\hat{\sigma }^{\prime} }_{x}\rangle$ (using the approximate Hamiltonian in Eq. (3)) (**a**) without and (**b**) with noise in the ultra strong system, after the coarse-graining measurements that projects the state into ${\hat{\rho }}_{x\ge 0}$ or ${\hat{\rho }}_{x < 0}$, depending on the measurement results. We set the coarse-graining value as $\sigma =5$. The noise rate ${\gamma }_{1}={\gamma }_{2}=2\pi \times 23.75\,{\rm{kHz}}$. For the other parameters, we use the same as those in Fig. 2 in the main text.

Here, $|k\rangle$ and $|j\rangle$ are the eigenstates of the system Hamiltonian and ${\kappa }_{q}(\omega )$ and ${\kappa }_{r}(\omega )$ are the rates corresponding to the noise spectra of the qubit and resonator, respectively. Also,

$${\Phi }_{j}^{{\rm{dep}}}=\sqrt{{\gamma }_{{\rm{dep}}}(0)/2}\langle j|{\hat{\sigma }}_{x}|j\rangle $$

and

$${\Gamma }_{{\rm{dep}}}^{jk}={\gamma }_{{\rm{dep}}}({\Delta }_{jk})/2|\langle j|{\hat{\sigma }}_{x}|k\rangle {|}^{2},$$

where ${\gamma }_{{\rm{dep}}}(\omega )$ denotes the spectral density of the qubit dephasing at frequency $\omega$. Here, we ignore the term ${\Gamma }_{{\rm{dep}}}^{jk} {\cal{D}} [|j\rangle \langle k|](\hat{\rho })$, as this term is negligible when we operate at the “sweet spot” of the qubit. Owing to the two-level approximation, we consider only the lowest first two levels $|G\rangle$ and $|E\rangle$, and defining ${\gamma }_{1}={\Gamma }_{{\sigma }_{z}}^{10}+{\Gamma }_{X}^{10}$, and ${\gamma }_{2}={({\Phi }_{0}^{{\rm{dep}}})}^{2}={({\Phi }_{1}^{{\rm{dep}}})}^{2}$, we obtain

$$\begin{array}{rcl}\frac{d\hat{\rho }}{dt} & = & -i[\hat{H}^{\prime} ,\hat{\rho }]+{\gamma }_{1} {\cal{D}} [|G\rangle \langle E|](\hat{\rho })+{\gamma }_{2} {\cal{D}} [|E\rangle \langle E|-|G\rangle \langle G|](\hat{\rho }),\\ \hat{H}^{\prime}  & = & \frac{{\omega }_{{\rm{q}}}}{2}\,\exp (\,-\,2{\alpha }^{2})(|E\rangle \langle E|-|G\rangle \langle G|),\end{array}$$

where $\alpha =g/{\omega }_{{\rm{r}}}$. In Fig. 13(a), we plot the expectation value of ${\hat{\sigma }}_{z}$ and ${\hat{\sigma }^{\prime} }_{x}=|E\rangle \langle G|+|G\rangle \langle E|$ without the noise in the ultra-strongly coupled system. The two-level approximation shows an excellent agreement with the full Hamiltonian model. Also, in Fig. 13(b), we plot ${\hat{\sigma }}_{z}$ and ${\hat{\sigma }^{\prime} }_{x}$ including the noise in the ultra-strongly-coupled system with parameters^70^ that are realized in recent experiments^71^. From these results, we can conclude that the noise in the ultra-strongly coupled system is almost negligible and does not have significance on the time scales in which we are interested.

### F. Detailed explanation of the AC Stark shift

We assume the regime where the time scale of the dynamics of photons in the nonlinear resonator is much smaller than $1/{\omega }_{{\rm{eff}}}$. In this case, we can approximate the interaction term $J{\hat{\sigma }^{\prime} }_{x}{\hat{b}}^{\dagger }\hat{b}\approx J{\hat{\sigma }^{\prime} }_{x}\langle {\hat{b}}^{\dagger }\hat{b}\rangle$, where $\langle {\hat{b}}^{\dagger }\hat{b}\rangle$ is the expectation value of the photon number in the nonlinear resonator.

Therefore, the approximated two-level system can be described with the effective Hamiltonian,

$${\hat{H}}_{{\rm{eff}}}=J\langle {\hat{b}}^{\dagger }\hat{b}\rangle {\hat{\sigma }^{\prime} }_{x}+\frac{{\omega }_{{\rm{eff}}}}{2}{\hat{\sigma }^{\prime} }_{z}.$$

Now, as the initial state is $|G\rangle$, and ${\langle {\hat{b}}^{\dagger }\hat{b}\rangle }_{t=0}=0$, the initial state is also the ground state of ${\hat{H}}_{{\rm{eff}}}$. Due to the assumption that $\langle {\hat{b}}^{\dagger }\hat{b}\rangle$ slowly changes compared with the evolution induced by ${\omega }_{{\rm{eff}}}$, the state stays at the ground state of ${\hat{H}}_{{\rm{eff}}}$.

Then, the nonlinear resonator can produce the mixed state due to the bifurcation as

$${\hat{\rho }}_{non}={p}_{{\rm{High}}}(t)|{\rm{High}}(t)\rangle \langle {\rm{High}}(t)|+{p}_{{\rm{Low}}}(t)|{\rm{Low}}(t)\rangle \langle {\rm{Low}}(t)|,$$

where $|{\rm{High}}(t)\rangle$ and $|{\rm{Low}}(t)\rangle$ are the high and low amplitude states at time *t*. Now, the approximated two-level system evolves according to $|{\rm{High}}(t)\rangle$ and $|{\rm{Low}}(t)\rangle$, which is a AC Stark shift. More concretely, the approximated two-level system birfurcates into two states: the ground states of ${\hat{H}}_{{\rm{eff}}}^{{\rm{High}}({\rm{Low}})}=J{\langle {\hat{b}}^{\dagger }\hat{b}\rangle }_{H(L)}{\hat{\sigma }^{\prime} }_{x}+\frac{{\omega }_{{\rm{eff}}}}{2}{\hat{\sigma }^{\prime} }_{z}$, where ${\langle {\hat{b}}^{\dagger }\hat{b}\rangle }_{H(L)}$ is the expectation value of the photon number corresponding to the high and low amplitude states. Consequently, the dynamics of the composite system of the approximated two-level system and the nonlinear resonator can be described as

$$\begin{array}{ccc}|G\rangle |0\rangle  & \to  & {p}_{{\rm{H}}{\rm{i}}{\rm{g}}{\rm{h}}}(t)|{G}^{({\rm{H}}{\rm{i}}{\rm{g}}{\rm{h}})}(t)\rangle \langle {G}^{({\rm{H}}{\rm{i}}{\rm{g}}{\rm{h}})}(t)|\otimes |{\rm{H}}{\rm{i}}{\rm{g}}{\rm{h}}(t)\rangle \langle {\rm{H}}{\rm{i}}{\rm{g}}{\rm{h}}(t)|\\  &  & +\,{p}_{{\rm{L}}{\rm{o}}{\rm{w}}}(t)|{G}^{({\rm{L}}{\rm{o}}{\rm{w}})}(t)\rangle \langle {G}^{({\rm{L}}{\rm{o}}{\rm{w}})}(t)|\otimes |{\rm{L}}{\rm{o}}{\rm{w}}(t)\rangle \langle {\rm{L}}{\rm{o}}{\rm{w}}(t)|,\end{array}$$

where $|{G}^{({\rm{High}}({\rm{Low}}))}(t)\rangle$ is the ground state of ${\hat{H}}_{{\rm{eff}}}$ for high and low amplitude states. Considering the case that the number of photons in the high-amplitude states is extremely high, with the low-amplitude state having approximately zero photon, $|{G}^{({\rm{H}}{\rm{i}}{\rm{g}}{\rm{h}})}(t)\rangle \approx |-\rangle \equiv \frac{1}{\sqrt{2}}(|G\rangle -|E\rangle )$, and $|{G}^{({\rm{Low}})}(t)\rangle \approx |G\rangle$.

Therefore, the post measured state corresponding to high and low amplitude states are $|\,-\,\rangle$ and $|G\rangle$. Thus, the results in Fig. 4 became asymmetric. In addition, the dynamics of the birfurcation crucially depends on the pumping strength (also the detuning, and the strength of the nonlinearlity). In some regime, a bifurcation does not appear, and the dynamics described above completely changes.

### G Quantum discord

To elucidate the previous results further, we consider the quantum discord (QD), which is defined as follows. Two possible definitions of the mutual information of the state ${\hat{\rho }}_{AB}$

$$\begin{array}{rcl}I({\hat{\rho }}_{AB}) & = & S({\hat{\rho }}_{A})+S({\hat{\rho }}_{B})-S({\hat{\rho }}_{AB})\\ {J}_{A}({\hat{\rho }}_{AB}) & = & S({\hat{\rho }}_{B})-S({\hat{\rho }}_{B}|{\hat{\rho }}_{A}),\end{array}$$

where $S(\hat{\rho })$ is the von Neumann entropy for a state $\hat{\rho }$, ${\hat{\rho }}_{A(B)}$ is the reduced density operator for ${ {\mathcal H} }_{A(B)}$, and $S({\hat{\rho }}_{B}|{\hat{\rho }}_{A})$ is the quantum generalization of the conditional entropy. In the purely classical case, one can show that these two definitions of the mutual information are equivalent. However, in the nonclassical case, these definitions do not necessarily coincide. Also, ${J}_{A}({\hat{\rho }}_{AB})$ is dependent on the measurement basis $\hat{M}$ for ${ {\mathcal H} }_{A}$. Therefore, the QD is defined as

$${\mathscr{Q}}=I({\hat{\rho }}_{AB})-{max}_{\hat{M}}\{{J}_{\hat{M}}({\hat{\rho }}_{AB})\}=S({\hat{\rho }}_{A})-S({\hat{\rho }}_{AB})+{min}_{\hat{M}}S({\hat{\rho }}_{B|\{\hat{M}\}}),$$

where

$$S({\hat{\rho }}_{B|\{\hat{M}\}})=\sum _{k}\,{p}_{k}S({\hat{M}}_{k}{\hat{\rho }}_{AB}{\hat{M}}_{k}/{p}_{k}),\,{p}_{k}={\rm{T}}{\rm{r}}({\hat{M}}_{k}\hat{\rho }).$$

Here ${\hat{M}}_{k}$ is a projector when the result is $k$, and the QD is basis independent and reflects only nonclassical correlations^72,73^. In our case, system $A$ corresponds to the approximated two-level system and system $B$ the nonlinear resonator. We set the measurement basis on the approximated two-level system as $\{|{\varphi }_{1}\rangle \langle {\varphi }_{1}|,|{\varphi }_{2}\rangle \langle {\varphi }_{2}|\}$,

$$\begin{array}{ccc}|{\varphi }_{1}\rangle  & = & \cos (\theta /2)|g\rangle +{e}^{i\phi }\,\sin (\theta /2)|e\rangle ,\\ |{\varphi }_{2}\rangle  & = & \sin (\theta /2)|g\rangle -{e}^{i\phi }\,\cos (\theta /2)|e\rangle ,\end{array}$$

$(0\le \theta \le \pi ,0\le \phi  < 2\pi )$, where $|e\rangle$ and $|g\rangle$ are the eigenstates of ${\hat{\sigma }^{\prime} }_{x}$. Given these definitions we find the $(\theta ,\phi )$ which realize ${min}_{\hat{M}}S({\hat{\rho }}_{B|\{\hat{M}\}})$.

**Figure 14:**
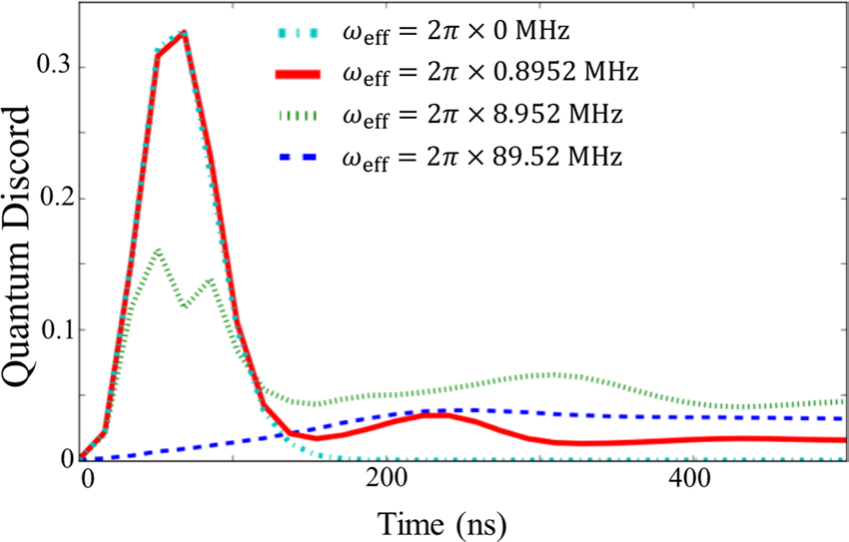
Quantum discord between the ultra-strongly coupled system and the nonlinear resonator. From the top, we plot results with effective energies ${\omega }_{{\rm{eff}}}=2\pi \times 0\,{\rm{MHz}}$, ${\omega }_{{\rm{eff}}}=2\pi \times 0.8952\,{\rm{MHz}}$, ${\omega }_{{\rm{eff}}}=2\pi \times 8.952\,{\rm{MHz}}$, and ${\omega }_{{\rm{eff}}}=2\pi \times 89.52\,{\rm{MHz}}$. Except for the effective energy of the ultra-strongly coupled system, here we use the same parameters as those in Fig. 4.

We plot the QD in Fig. 14. Interestingly, in contrast to the negativity, the QD, at $t=500\,{\rm{ns}}$, becomes larger as $J/{\omega }_{{\rm{q}}}$ is decreased. This can be explained in the following way: if $J/{\omega }_{{\rm{q}}}$ is sufficiently large, the state becomes a highly entangled state well approximated by the form

$$\frac{1}{\sqrt{2}}(|e\rangle |{\rm{Low}}\rangle -|g\rangle |{\rm{High}}\rangle ),$$

which decays, due the measurement of the nonlinear cavity, to the mixture

$${\hat{\rho }}_{{\rm{f}}}=\frac{1}{2}(|e\rangle \langle e|\otimes |{\rm{Low}}\rangle \langle {\rm{Low}}|+|g\rangle \langle g|\otimes |{\rm{High}}\rangle \langle {\rm{High}}|),$$

where $|{\rm{High}}\rangle$ and $|{\rm{Low}}\rangle$ are high and low amplitude states of the nonlinear resonator. Since $|e\rangle$ and $|g\rangle$ are orthogonal to each other, ${\hat{\rho }}_{{\rm{f}}}$ is a classically correlated state without any superposition, implying the vanishing QD. On the other hand, when $J/{\omega }_{{\rm{q}}}$ is small, the dynamics can be explained by an AC Stark shift and the state can be expressed as

$$\begin{array}{rcl}{\hat{\rho }}_{{\rm{a}}} & = & {p}_{{\rm{High}}}|{G}^{({\rm{High}})}\rangle \langle {G}^{({\rm{High}})}|\otimes |{\rm{High}}\rangle \langle {\rm{High}}|\\  &  & +\,{p}_{{\rm{Low}}}|{G}^{({\rm{Low}})}\rangle \langle {G}^{({\rm{Low}})}|\otimes |{\rm{Low}}\rangle \langle {\rm{Low}}|,\end{array}$$

where ${p}_{{\rm{Low}}({\rm{High}})}$ is the probability that the nonlinear resonator is in the low (or high) amplitude state. The state $|{G}^{({\rm{High}}({\rm{Low}}))}\rangle$ is the ground state of ${\hat{H}}_{{\rm{eff}}}$. Here, $|{G}^{({\rm{High}})}\rangle$ and $|{G}^{({\rm{Low}})}\rangle$ are not always orthogonal to each other, and, as such, the correlation in the mixture of the two could have a non-classical nature. Hence, the QD, in the long-time limit, tends to have a finite value when *J*/${\omega }_{{\rm{q}}}$ is small.
